# Oscillating systems with cointegrated phase processes

**DOI:** 10.1007/s00285-017-1100-2

**Published:** 2017-01-30

**Authors:** Jacob Østergaard, Anders Rahbek, Susanne Ditlevsen

**Affiliations:** 1 Department of Mathematical Sciences, University of Copenhagen, Universitetsparken 5, 2100 Copenhagen Ø, Denmark; 2 Department of Economics, University of Copenhagen, Øster Farimagsgade 5, Building 26, 1353 Copenhagen K, Denmark

**Keywords:** Coupled oscillators, Synchronization, Cointegration, Phase process, Interacting dynamical system, Winfree oscillator, EEG signals, 37N25, 62M10, 92B25, 62F03

## Abstract

We present cointegration analysis as a method to infer the network structure of a linearly phase coupled oscillating system. By defining a class of oscillating systems with interacting phases, we derive a data generating process where we can specify the coupling structure of a network that resembles biological processes. In particular we study a network of Winfree oscillators, for which we present a statistical analysis of various simulated networks, where we conclude on the coupling structure: the direction of feedback in the phase processes and proportional coupling strength between individual components of the system. We show that we can correctly classify the network structure for such a system by cointegration analysis, for various types of coupling, including uni-/bi-directional and all-to-all coupling. Finally, we analyze a set of EEG recordings and discuss the current applicability of cointegration analysis in the field of neuroscience.

## Introduction

Since the first scientific discovery of two pendulums synchronizing by Christiaan Huygens in the seventeenth century, this naturally occurring phenomenon has now been observed in diverse areas such as fireflies synchronizing their flashing behavior, a theatre audience applauding after a show and also in chemical and biological systems, such as the brain and the heart beats of a mother and her fetus, where coupled oscillators appear, see also Pikovsky et al. (2001). Due to it’s pervasive presence, understanding synchronization is of key interest for researchers to understand biological networks, such as the connectivity of the nervous system, circadian rhythms or the cardiovascular system. To a statistician this presents a fascinating challenge of modelling complex behavior in large scale systems and how to infer the data-generating mechanisms. To this day, synchronization is not fully understood, but has been the centre of research for decades as evident in Ermentrout (1985), Kuramoto (1984), Strogatz (1987, 2000), Taylor and Holmes (1998), Winfree (1967), even the phenomenon of synchronizing pendulums as observed by Huygens, still attracts attention today, see Martens et al. (2013), Oliveira and Melo (2015). Many innovative ideas have been presented since Winfree (1967) began a mathematical treatment of the subject. When Kuramoto (1984) first presented his model of coupled oscillators, this made a huge impact in the field and spawned a new generation of research on synchronization. Kuramotos model is still considered among one of the most significant advancements in the study of synchronization in oscillating systems as acknowledged by Strogatz (2000), and the study of coupled oscillators still attracts a fair interest from researchers Ashwin et al. (2016), Burton et al. (2012), Fernandez and Tsimring (2014), Ly (2014), Ly and Ermentrout (2011).

A long standing problem in neuroscience is to recover the network structure in a coupled system. This could for example be to infer the functional connectivity between units in a network of neurons from multiple extracellularly recorded spike trains, or how traces of EEG signals from different locations on the scalp affect each other, which we will treat in this paper. To the authors knowledge, this challenge is still lacking a sound statistical framework to model and test for interaction in a system, as well as impose statistical hypotheses on the network structure. For this task, cointegration analysis offers a refined statistical toolbox, where detailed information on the connections can be inferred, such as the direction and proportional strength of the coupling. The theory of cointegration was originally conceived by Granger (1981), and has since then also been the subject of intense research, most notably within the field of econometrics. In the monograph by Johansen (1996), the full likelihood theory for linear cointegration models with Gaussian i.i.d. errors is derived, and a framework for estimation and inference on parameters using the quotient test is presented. This well acknowledged framework is popularly termed the Johansen procedure. Even though cointegration analysis has developed from within the field of econometrics, it may potentially be used for different models outside economics, such as biological models in continuous time as we explore here. It has also been applied in climate analysis, see Schmith et al. (2012).

In this paper, we demonstrate how to apply cointegration analysis to a system of linearly phase coupled oscillating processes. To display the applicability of the method, we present a simulation experiment, where we present a statistical analysis of phase coupled systems with varying network structures, including uni-/ bi-directional and all-to-all couplings. We show that we can identify the proportional coupling strengths and directions given by the estimated *cointegration matrix* parameter. Our work is inspired by Dahlhaus and Neddermeyer (2012), which also introduces cointegration analysis as a statistical toolbox to neuroscientists and new challenges for researchers in cointegration theory. However, in contrast to Dahlhaus and Neddermeyer (2012), we incorporate the fact that we are dealing with continuous systems and also ensure that the cointegration property of the system is well posed as a linear structure. This approach assures that the conclusion on the interaction in the data is accurate in terms of cointegration.

The paper is composed as follows. In Sect. 2 we define a class of phase coupled oscillators, in Sect. 3 we highlight some cointegration theory for the analysis including an extension to discretely observed, continuous time models. In Sect. 4 we present a statistical analysis of linearly phase coupled oscillating systems and in Sect. 5 we analyze EEG recordings from an epileptic subject experiencing a seizure, previously analyzed by Shoeb (2009). We discuss the model and findings, conclude on the research and give an outlook of the future direction of the research in Sect. 6. Technical details are presented in the appendix.

Throughout we use the following notation and conventions: unless explicitly stated otherwise, time $t\in [0,\infty )$ is assumed continuous, and the process $(x_t,y_t)'$ is assumed observed with corresponding polar coordinates $(\phi _t,\gamma _t)'$. Here $'$ denotes transposition. For a $p\times r$ matrix *M*, with $r\le p$, we denote the orthogonal complement $M_\perp$, a $p \times (p-r)$ matrix such that $M'_\perp M=0$ (zero matrix). Also denote by $\text {sp}(A)$ the subspace spanned by the columns of a matrix *A*, and let ${{\mathrm{rank}}}(A)$ denote the rank of the matrix, i.e., the dimension of $\text {sp}(A)$.

## Oscillating systems

Studying biological rhythms corresponds to studying systems of periodical processes. Intuitively we define a single *oscillator* as a continuous time bi-variate process $z_t = (x_t,y_t)'\in {\mathbb {R}}^2$, $t\in [0,\infty )$, such that $z_t$ revolve around some arbitrary center. Such a process can be derived from an equivalent process in polar coordinates $(\phi _t,\gamma _t)'$, where $\phi _t\in {\mathbb {R}}$ is the *phase* process and $\gamma _t\in {\mathbb {R}}$ is the *amplitude* process, such that

1 $$\begin{aligned} \begin{aligned} x_t&= \gamma _t\cos (\phi _t)\\ y_t&= \gamma _t\sin (\phi _t). \end{aligned} \end{aligned}$$

We then define the process $z_t$ to be an oscillator if the phase process has a monotonic trend.

### Defining a class of coupled oscillators

Definition (1) naturally extends to a system of *coupled stochastic oscillators*, where we observe *p* oscillators that interact, i.e., $z_t\in {\mathbb {R}}^{2p}$. Define a class of oscillators with phase ($\phi _t\in {\mathbb {R}}^p$) and amplitude ($\gamma _t\in {\mathbb {R}}^p$) processes given by the multivariate stochastic differential equations (SDE)

2 $$\begin{aligned} d\phi _t&= f(\phi _t,\gamma _t)dt+\varSigma _\phi dW^\phi _t \end{aligned}$$

3 $$\begin{aligned} d\gamma _t&= g(\phi _t,\gamma _t)dt+\varSigma _\gamma dW^\gamma _t, \end{aligned}$$

where $f,g:{\mathbb {R}}^{2p}\rightarrow {\mathbb {R}}^p$ are real valued vector functions, possibly depending on both $\phi _t,\gamma _t$ or constant, $dW_t^\phi ,dW_t^\gamma$ are multivariate standard Wiener processes and $\varSigma _\phi ,\varSigma _\gamma \in {\mathbb {R}}^{p\times p}$ such that $\varSigma _i\varSigma _i'$ is a positive semi-definite covariance matrix for $i=\phi ,\gamma$. Assume the properties of (2) and (3) are such that

4 $$\begin{aligned} \gamma _t\in {\mathbb {R}}^p_+ \quad \text {for } t\in [0,\infty ) \end{aligned}$$

and

5 $$\begin{aligned} \mathbb {E}[\phi _{kt}] \text { is monotonically increasing as a function of } t \text { for each } k=1,\ldots ,p, \end{aligned}$$

where $\mathbb {E}[\cdot ]$ denotes the mean. Since $\gamma _t = (\gamma _{1t},\ldots , \gamma _{pt})'$ are interpreted as the amplitudes of the individual oscillators, Eq. (4) is a natural assumption and Eq. (5) ensures that the individual oscillators actually revolve (anti-clockwise) around the center and that they are not ”stuck” in some part of the phase space, i.e., their *angular velocities* are positive. Note that we have defined the phase-trend as positive, corresponding to counter-clockwise rotation in accordance with the standard interpretation of the phase. However, for a negative trending process, one can either look at $-\phi _t$ or simply interpret rotations as clockwise.

To emphasize the implication of inducing interaction in a system, for the data generating process (DGP) in the *xy*-plane, we derive a DGP from (2)–(3), see “Appendix 1”. Assuming that $\varSigma _\phi = {{\mathrm{diag}}}(\sigma _1^\phi ,\ldots ,\sigma _p^\phi )$ and $\varSigma _\gamma = {{\mathrm{diag}}}(\sigma _1^\gamma ,\ldots ,\sigma _p^\gamma )$ we find that

6 $$\begin{aligned} d\begin{pmatrix} x_{kt} \\ y_{kt} \end{pmatrix}&= \begin{pmatrix} -\frac{1}{2}\left( \sigma _k^\phi \right) ^2 &{} \quad -f_k(\phi _t,\gamma _t) \\ f_k(\phi _t,\gamma _t) &{} \quad -\frac{1}{2}\left( \sigma _k^\phi \right) ^2 \end{pmatrix} \begin{pmatrix} x_{kt} \\ y_{kt} \end{pmatrix}dt+ \begin{pmatrix} 0 &{}\quad -\sigma _k^\phi \\ \sigma _k^\phi &{}\quad 0 \end{pmatrix} \begin{pmatrix} x_{kt} \\ y_{kt} \end{pmatrix}dW_{kt}^\phi \nonumber \\&\quad +\frac{g_k(\phi _t,\gamma _t)+\sigma _k^\gamma \sigma _k^\phi }{\sqrt{x_{kt}^2+y_{kt}^2}} \begin{pmatrix} x_{kt} \\ y_{kt} \end{pmatrix}dt+ \frac{\sigma _k^\gamma }{\sqrt{x_{kt}^2+y_{kt}^2}} \begin{pmatrix} x_{kt} \\ y_{kt} \end{pmatrix}dW_{kt}^\gamma . \end{aligned}$$

Hence, with the definitions (2)–(5) we have introduced a general class of coupled oscillators, where the specifications of *f* and *g* define the properties of the system, such as interaction. This broad definition of oscillating systems covers among others the Kuramoto model, see example (Sect. 2.5) below and other standard oscillators such as the FitzHugh–Nagumo and the Duffing oscillator. In this paper we will analyze *phase coupled* oscillators, and therefore we assume that $g_k(\phi _t,\gamma _t)=g_k(\gamma _{kt})$, such that there is no feedback from the phase process $\phi _t$ into the amplitude and the *k*’th amplitude is not dependent on the rest. Hence, interaction in the system is solely through $f(\phi _t,\gamma _t)$, such that the phase processes are attracted by some interdependent relation.

### Linear coupling

The arbitrary function *f* enables us to choose any transformation of the variables to obtain a coupled system, including unidirectional coupling between phases or periodic forcing of the system if we extend *f* to depend on *t* as well, intermittent synchronization dependent on a threshold in process differences, etc.

Studying the general case where $f(\phi _t,\gamma _t)$ is nonlinear in $\phi _t$ and $\gamma _t$ is a complex exercise. In this paper we restrict ourselves to models where *f* is composed of a linear mapping of $\phi _t$ and a function of $\gamma _t$, with components,

7 $$\begin{aligned} f_k(\phi _t,\gamma _t) = \sum _{j=1}^p\varPi _{kj}(\phi _{jt}-\omega _j)+h(\gamma _{kt}),\quad \text {for }k=1,\ldots ,p \end{aligned}$$

for a real matrix $\varPi \in {\mathbb {R}}^{p\times p}$ and constant vector $\omega =(\omega _1,\ldots ,\omega _p)'\in {\mathbb {R}}^p$. With this restriction, the interaction between oscillators is linear in the phase, and the *k*’th oscillator is only dependent on the intrinsic amplitude $\gamma _{kt}$ through $h(\gamma _{kt})$. We will refer to such a system as *linearly phase coupled*.

Although we impose the linear restriction $\varPi$ on the interaction between phases, we can still model a broad set of coupling structures as we show with examples below. Since the interaction is given by $\varPi \phi _t$, we note that the *coupling strength* in the system is given as the absolute values of the entries of $\varPi$ and that row *k* of $\varPi$ define how oscillator *k* depends on the rest. Note also that $\omega$ defines the attracting state for the phase relations, see example (Sect. 2.3) below. Normally $h(\gamma _{kt})$ is restricted to a constant, but in Sect. 4 we will relax this and investigate systems where $h(\gamma _{kt})$ is only approximately linear and has a sufficiently low variance. This implies a misspecified model, but as we will show, we can still identify the coupling structure, although inference on $h(\gamma _{kt})$ itself is less meaningful.

### Example: Linearly phase coupled system with a degenerate $\gamma _t$ process

Let *f* be defined as in (7) and assume that $\gamma _t$ is a constant (positive) process such that $h(\gamma _{kt})=\mu _k>0$. Then *f* is of the form

8 $$\begin{aligned} f(\phi _t)=\varPi (\phi _t-\omega )+\mu , \end{aligned}$$

where $\omega ,\mu \in {\mathbb {R}}^p$ are constant vectors. For reduced rank matrices $\varPi$ (2) is a continuous time cointegrated process (see Sect. 3) and *f* admits a linearly phase coupled system with intrinsic rotating frequencies $\mu$. Note that if $\varPi =0$ then there is no interaction in the system, and the individual oscillators will rotate according to their own $\mu _k>0$, and we refer to the system as *independent*.

The linear specification $\varPi (\phi _t-\omega )$ implies that at most one attracting point can exist. As an illustration of this, assume a system composed of two coupled oscillators, with

$$\begin{aligned} \varPi (\phi _t-\omega ) = \begin{pmatrix} -\alpha _1 &{} \quad &{}\,\alpha _1 \\ \,\alpha _2 &{} \quad &{} -\alpha _2 \end{pmatrix}\begin{pmatrix} \phi _{1t}-\omega _1 \\ \phi _{2t}-\omega _2 \end{pmatrix} = \begin{pmatrix} -\alpha _1 \\ \,\alpha _2 \end{pmatrix}\Bigl ((\phi _{1t}-\phi _{2t})-(\omega _1-\omega _2)\Bigr ). \end{aligned}$$

where $0<\alpha _1+\alpha _2<2$. Since $\omega ^*=\omega _1-\omega _2$ define an attracting state of the phase difference $\phi _{1t}-\phi _{2t}$, then with $\omega ^*=0$ the system is attracted towards being *in-phase*, whereas $\omega ^*=\pi$ would imply that the system is attracted towards being in *anti-phase*. Considering that neither $\alpha _1,\alpha _2$ or $\omega ^*$ depend on time, the system cannot switch to a different attracting regime.

To illustrate possible coupling structures, consider again the system of two oscillators and assume that $\omega =0$. Then with $\alpha _2=0$ and $\alpha _1\ne 0$ the coupling between $\phi _{1t},\phi _{2t}$ is *uni-directional* $\phi _{2t}\rightarrow \phi _{1t}$ where the arrow $\rightarrow$ denote the direction of interaction. Likewise, if $\alpha _1=0$ and $\alpha _2\ne 0$ then $\phi _{1t}\rightarrow \phi _{2t}$. However, if both $\alpha _1,\alpha _2\ne 0$ then $\phi _{2t}\leftrightarrow \phi _{1t}$ and the coupling is *bi-directional*. In general, if $\phi _{kt}$ appears in the expression $f_l(\phi _t)$ for oscillator $l\ne k$, then $\phi _{kt}\rightarrow \phi _{lt}$. If the opposite is true, then $\phi _{lt}\rightarrow \phi _{kt}$ and if both directions exist, then $\phi _{lt}\leftrightarrow \phi _{kt}$. For $f_k(\phi _t)=0$ oscillator *k* is (oneway) independent from the rest, but it can still possibly influence others.

For systems where $\gamma _t$ is a degenerate process, then $\varSigma _\gamma =0$ and $g(\phi _t,\gamma _t)=0$. With $\sigma _k^\phi =\sigma _k$ then (6) simplifies to

9 $$\begin{aligned} d\begin{pmatrix} x_{kt} \\ y_{kt} \end{pmatrix} = \begin{pmatrix} -\frac{1}{2}\sigma _k^2 &{} \quad -f_k(\phi _t) \\ f_k(\phi _t) &{} \quad -\frac{1}{2}\sigma _k^2 \end{pmatrix} \begin{pmatrix} x_{kt} \\ y_{kt} \end{pmatrix}dt+\begin{pmatrix} 0 &{}\quad -\sigma _k \\ \sigma _k &{}\quad 0 \end{pmatrix} \begin{pmatrix} x_{kt} \\ y_{kt} \end{pmatrix}dW_k, \end{aligned}$$

where $f_k(\phi _t)=\sum _j\varPi _{kj}\phi _{jt}+\mu _k$. Note that if $\varPi = 0$ then (8) is simply a constant trend and hence (9) is a rotating process. One can show that the eigenvalues of the deterministic drift matrix in (9) in this case are complex conjugates, $-\frac{\sigma ^2}{2}\pm i\mu$, where $i=\sqrt{-1}$, implying that the solutions to (9) oscillate for $\mu \ne 0$. The oscillations are damped by the negative real part, but sustained by the noise term.

When $\gamma _t$ is a constant vector process the properties of the system are fully identified by (2). Furthermore, if the noise level of the phases $\varSigma _\phi$ is sufficiently small, we can use the Hilbert transform^1^ to derive the phase process $\phi _t$ from observations of either $x_t$ or $y_t$. This is a commonly used technique in signal processing and has been applied to oscillating systems as well, see Dahlhaus and Neddermeyer (2012), Pikovsky et al. (2001). For systems where $\phi _t$ is very noisy, this method is less applicable.

### Example: Winfree oscillator

Let $g_k(\phi ,\gamma ) = (\kappa _k-\gamma _k)\gamma _k^2$ for a vector $\kappa \in {\mathbb {R}}_+^p$ and $f_k(\phi ,\gamma ) = \sum _{j=1}^p\varPi _{kj}\phi _j+\gamma _k$ for $\varPi \in {\mathbb {R}}^{p\times p}$ such that

$$\begin{aligned} d\gamma _{kt}&= (\kappa _k-\gamma _{kt})\gamma _{kt}^2dt+\sigma _k^\gamma dW_{kt}^\gamma \\ d\phi _{kt}&= \Bigl ( \sum _{j=1}^p\varPi _{kj}\phi _j+\gamma _{kt}\Bigr )dt +\sigma _k^\phi dW_{kt}^\phi . \end{aligned}$$

With these definitions (6) becomes

10 $$\begin{aligned} d\begin{pmatrix} x_{kt} \\ y_{kt} \end{pmatrix}&= \begin{pmatrix} (\kappa _k-\gamma _{kt})\gamma _{kt}+\gamma _{kt}^{-1}\sigma _k^\gamma \sigma _k^\phi -\frac{1}{2}\left( \sigma _k^\phi \right) ^2 &{} \quad -\Bigl (\sum _{j=1}^p\varPi _{kj}\phi _j+\gamma _{kt}\Bigr ) \\ \Bigl (\sum _{j=1}^p\varPi _{kj}\phi _j+\gamma _{kt}\Bigr ) &{} \quad (\kappa _k-\gamma _{kt})\gamma _{kt}+\gamma _{kt}^{-1}\sigma _k^\gamma \sigma _k^\phi -\frac{1}{2}\left( \sigma _k^\phi \right) ^2 \end{pmatrix} \begin{pmatrix} x_{kt} \\ y_{kt} \end{pmatrix}dt \nonumber \\&\quad + \begin{pmatrix} 0 &{}\quad -\sigma _k^\phi \\ \sigma _k^\phi &{}\quad 0 \end{pmatrix} \begin{pmatrix} x_{kt} \\ y_{kt} \end{pmatrix} dW_{kt}^\phi +\begin{pmatrix} \gamma _{kt}^{-1}\sigma _k^\gamma &{}\quad 0 \\ 0 &{} \quad \gamma _{kt}^{-1}\sigma _k^\gamma \end{pmatrix} \begin{pmatrix} x_{kt} \\ y_{kt} \end{pmatrix} dW_{kt}^\gamma . \end{aligned}$$

This example is taken from Winfree (2001) and extended with noise and phase interaction, and therefore we will refer to (10) as the (noisy) *Winfree oscillator*. Note that the formulation of $d\gamma _{kt}$ implies that the amplitude fluctuates around $\kappa _k$. Due to this, we can for sufficiently small noise $\varSigma _\gamma$ insist that $\gamma _{kt} \approx \kappa _k$ for $k=1,\ldots ,p$ and therefore analyze the Winfree oscillator using the cointegration toolbox, assuming a constant $\gamma _{t}$ in $d\phi _t$. In Sect. 4 we analyze the range of noise, $\varSigma _\gamma$, where the cointegration analysis still performs well.

### Example: Kuramoto model

Choose $f(\phi _t,\gamma _t)$ such that

11 $$\begin{aligned} f_k(\phi _t,\gamma _t)=f_k(\phi _t) = \frac{1}{p}\sum _{j=1}^p K_{kj}\sin (\phi _{jt}-\phi _{kt})+\mu _i,\quad k=1,\ldots ,p, \end{aligned}$$

then (2) is the Kuramoto model extended with a stochastic noise term, for phase coupled oscillators, where $K_{kj}$ denotes the coupling strength between the *k*’th and *j*’th oscillators. In the classic version, $K_{kj}=K$ $\forall k,j$, such that for a certain threshold $K_c$, then with $K > K_c$ the oscillators exhibit synchronization. For an arbitrary $\gamma _t$ process we cannot simplify (6), but with a degenerate $\gamma _t$ we obtain the same expression as in (9) with $f_k(\phi _t)$ as in (11).

For the Kuramoto model *f* is a nonlinear function, hence it is not directly applicable to a standard cointegration analysis where *f* is assumed linear. To emphasize this fact, consider the special case $p=2$, where the Kuramoto model is particularly simple and (11) can be written explicitly as,

$$\begin{aligned} f(\phi _t)&= \frac{1}{2}\begin{pmatrix} \alpha _1\sin (\phi _{2t}-\phi _{1t}) \\ \alpha _2\sin (\phi _{1t}-\phi _{2t}) \end{pmatrix} +\mu = \frac{1}{2}\begin{pmatrix} -\alpha _1 \\ \,\alpha _2 \end{pmatrix} \sin (\phi _{1t}-\phi _{2t}) +\mu \\&= \frac{1}{2} \begin{pmatrix} -\alpha _1 \\ \,\alpha _2\end{pmatrix} \sin (\beta '\phi _t) +\mu . \end{aligned}$$

where $\beta ' = (1,-1)$ and $(\alpha _1,\alpha _2) = (K_{12},K_{21})$. If $\phi _{1t}\approx \phi _{2t}$ at $t=0$ and the values of $\alpha _1,\alpha _2$ are large enough, then $\phi _{1t}\approx \phi _{2t}\forall t$, such that $\beta '\phi _t\approx 0$ and we can write a crude linear approximation of the sine function: $\sin (\beta '\phi _t)\approx \beta '\phi _t$, such that

12 $$\begin{aligned} \begin{aligned} f(\phi _t)&\approx \frac{1}{2}\begin{pmatrix} -\alpha _1 \\ \,\alpha _2\end{pmatrix}\beta '\phi _t +\mu = \frac{1}{2} \begin{pmatrix} -\alpha _1 &{} \quad &{}\,\alpha _1 \\ \,\alpha _2 &{} \quad &{} -\alpha _2 \end{pmatrix} \phi _t +\mu . \end{aligned} \end{aligned}$$

This is a coarse, but linear, approximation of the Kuramoto model and we can perform a cointegration analysis assuming this approximation is satisfactory. However, one must be cautious with this approximation. Consider $\sin (\beta '\phi _t)$, when $\beta '\phi _t = \phi _{1t}-\phi _{2t} \approx \pi$. In this case $\sin (\beta '\phi _t)\approx \pi -\beta '\phi _t$, and hence

13 $$\begin{aligned} \begin{aligned} f(\phi _t)&\approx \frac{1}{2} \begin{pmatrix} -\alpha _1 \\ \,\alpha _2\end{pmatrix} (\pi -\beta '\phi _t) +\mu = \frac{1}{2} \begin{pmatrix} \,\alpha _1 &{} \quad &{} -\alpha _1 \\ -\alpha _2 &{} \quad &{} \,\alpha _2 \end{pmatrix} \phi _t +\mu + \begin{pmatrix} -\alpha _1 \\ \,\alpha _2\end{pmatrix}\pi , \end{aligned} \end{aligned}$$

and we see that not only do we add a term with $\pi$, but the interaction also reverses sign. Recall that $0<\alpha _1+\alpha _2<2$ which implies a stationary relation in the system in (12), see Sect. 3.2. In (13) this condition is reversed, in the sense that $-2<\alpha _1+\alpha _2<0$ will imply stationarity. If $0<\alpha _1+\alpha _2<2$, (13) leads to an explosive system, which is not covered in this paper. Therefore, an essential requirement for an approximation of the Kuramoto model is a regime switching ability of (2). For a model with this property, we propose that cointegration analysis on a piecewise linear approximation of the Kuramoto model does make sense and can lead to correct conclusions regarding the network structure. In this paper we will not deal with non-linear cointegration of oscillating systems, but leave this direction open for future research. For a statistical analysis of nonlinear cointegrated systems of the form $\alpha _t\beta '$, i.e. time varying, or regime switching $\alpha$ coefficients, see Bec and Rahbek (2004) and Kristensen and Rahbek (2013).

Note that with a general coupling constant $K_{kj}=K$, then the simple linear approximation to the Kuramoto model around $\phi _{jt}-\phi _{kt}\approx 0$ is

14 $$\begin{aligned} \frac{K}{p}\sum _{j=1}^p \begin{pmatrix} \sin (\phi _{jt}-\phi _{1t}) \\ \vdots \\ \sin (\phi _{jt}-\phi _{pt}) \end{pmatrix}&\approx \frac{K}{p} \begin{pmatrix} -(p-1) &{}\quad \dots &{}\quad 1 \\ \vdots &{}\quad \ddots &{}\quad \vdots \\ 1 &{}\quad \dots &{}\quad -(p-1) \\ \end{pmatrix}\phi _t. \end{aligned}$$

## Cointegration

Cointegration theory was originally developed for discrete time processes, however the ubiquitous use of continuous time models has inspired development of continuous time cointegration theory, see Kessler and Rahbek (2004, 2001). In order to present cointegration analysis as a framework for phase-processes, we therefore review some background on *discrete* time processes before entering into continuous time cointegrated models. The first part of this section is based on Johansen (1996) and Ltkepohl (2005).

### Integrated process

Assume that $\phi _n$ is a discrete time vector autoregressive process,

15 $$\begin{aligned} \phi _n = A\phi _{n-1}+\mu +\varepsilon _n, \end{aligned}$$

where $A\in {\mathbb {R}}^{p\times p}$, $\varepsilon _n$ is a Gaussian white noise and $\mu \in {\mathbb {R}}^p$ is a deterministic term. The *characteristic polynomial* for (15) is the determinant of $I_p-A\zeta$ for $\zeta \in {\mathbb {C}}$, where $I_p$ is the *p*-dimensional identity matrix. If the roots of the characteristic polynomial are all outside the unit circle, then the initial values of $\phi _n$ can be given a distribution such that $\phi _n$ is stationary, see Johansen (1996).

If the characteristic polynomial of (15) contains one or more roots at $\zeta =1$, then there is no stationary solution of $\phi _n$, and we say that the process is *integrated*. In particular, see Johansen (1996), $P=A-I_p$ will have reduced rank $r<p$ and can be written as $P= ab'$ with $a,b(p\times r)$ matrices of rank *r*. Moreover, the process $\phi _n$ is integrated of order one, *I*(1) with *r* cointegrating relations $b'\phi _n$ under regularity conditions presented in Sect. 3.2. Note that the order of integration is a stochastic property and hence including deterministic terms in a model does not change the order of integration.

In this paper we will only deal with *I*(1) processes, so when we refer to $\phi _n$ as integrated, we implicitly mean that $\phi _n$ is integrated of order 1.

### Cointegrated process

Let $\phi _n=\left( \phi _{1n},\ldots ,\phi _{pn}\right) ' \in R^{p}$ and rewrite (15) with $P=A-I_{p}$ as

16 $$\begin{aligned} \varDelta \phi _{n}=P\phi _{n-1}+\mu +\varepsilon _{n}. \end{aligned}$$

As already noted if $\det ( I-A\zeta ) =0$ implies $|\zeta |>1$ then $\phi _{n}$ has a stationary representation (as an *I*(0) process). In particular, *P* has full rank *p* and all linear combinations of $\phi _n$ are stationary. If the $(p\times p)$-dimensional matrix *P* has reduced rank $r<p$ then $P =ab'$ with $a,b,p\times r$ dimensional matrices of rank *r*. Moreover, the process $\phi _n$ is integrated of order one, *I*(1) with *r* cointegrating stationary relations $b'\phi _n$ provided $\rho (I_{r}+b'a) <1$ with $\rho \left( \cdot \right)$ denoting the spectral radius. This we refer to as the *I*(1) conditions in the following.

Note that if $r=0$ the process $\phi _n$ is *I*(1) with no cointegration, while if $r=p$ (and $\rho (A) <1$) then $\phi _n\,$is *I*(0), or *p* stationary linear combinations exist. Under the reduced rank *r*, the system is written as,

$$\begin{aligned} \varDelta \phi _{n}=ab^{\prime }\phi _{n-1}+\mu +\varepsilon , \end{aligned}$$

with *b* containing the *r* cointegration vectors and *a* the *loadings* or *adjustment coefficients*. Note that the entries of *a* and *b* are not uniquely identified, since we can use any non-singular transformation to obtain similar results. Rather we identify the subspaces $\text {sp}(a),\text {sp}(b)\in {\mathbb {R}}^r$, that is, the subspaces spanned by the columns of *a*, *b*, where we use the normalization

$$\begin{aligned} b^* = b(c'b)^{-1}, \quad \text {with } c = (I_r, 0_{p-r\times r})' \end{aligned}$$

of *b* in order to identify parameters uniquely. Furthermore, let $m_\perp$ denote the matrix such that $\text {sp}(m_\perp )$ is orthogonal to $\text {sp}(m)$, then a necessary condition for an *I*(1) process is that $|a_\perp 'b_\perp |\ne 0$. For more on estimation and inference in cointegration models, see “Appendix 2”.

### Continuous time cointegrated models

Kessler and Rahbek (2001, 2004) derive a cointegration theory for continuous time models, and conclude that for a discretely observed process, using conventional methods for discrete time generally apply to inference on continuous time parameters. Consider (2) with *f* as in (8) and for simplicity $\omega =0$. This is a *p*-dimensional Ornstein–Uhlenbeck process. The exact solution is

17 $$\begin{aligned} \phi _t&= \exp (t\varPi )\Bigl [\phi _0+\int _0^t \exp (-s\varPi )\mu ds+ \int _0^t\exp (-s\varPi )\varSigma dW_s\Bigr ]. \end{aligned}$$

Note that for the solution (17) to be stationary, then $\varPi$ must be a full rank matrix, and all eigenvalues must have a strictly negative real part. This implies that if $\varPi$ is *not* of full rank, then $\phi _t$ is necessarily not stationary.

Assuming discrete observations of (17) at equidistant timepoints $t_1=0<t_2<\cdots <t_N=T$ with timestep $\delta = t_{n}-t_{n-1}$, the corresponding vector autoregressive process is

18 $$\begin{aligned} \phi _{t_n} = \exp (\delta \varPi )\phi _{t_{n-1}}+\delta \mu +\varepsilon _{t_n}, \end{aligned}$$

such that the difference process can be written as

$$\begin{aligned} \varDelta \phi _{t_n} = \phi _{t_n}-\phi _{t_{n-1}}&= \delta P\phi _{t_{n-1}}+\delta \mu +\varepsilon _{t_n}, \end{aligned}$$

where $$\varepsilon \sim \mathcal {N}(0,\varOmega )$$ and

19 $$\begin{aligned} \begin{aligned} P&= \delta ^{-1}(\exp (\delta \varPi )-I_p) \\ \varOmega&= \int _0^{\delta } \exp (s\varPi )\varSigma \varSigma '\exp (s\varPi ')ds. \end{aligned} \end{aligned}$$

Results (18) and (19) hold in general for multivariate processes. Thus, to obtain an estimate for the continuous time matrix, $\hat{\varPi }$, from the discrete time estimate $\hat{P}$, a logarithmic transformation involving $\hat{P}$ is required

20 $$\begin{aligned} \hat{\varPi } = \delta ^{-1}\log \big (\delta \hat{P}+I_p\big ). \end{aligned}$$

For a univariate process (20) is unique, however this is not the case for a multivariate process, due to the non-uniqueness of the multivariate logarithm. Because of this, we cannot uniquely identify $\hat{\varPi }$, even though we have a unique estimate $\hat{P}$.

For a continuous time process $\phi _t$, however, Kessler and Rahbek (2001, 2004) conclude that this is cointegrated if and only if the discretely observed process (18) is cointegrated. In this case *P* is of reduced rank, and can be decomposed $P=ab'$ with $a,b\in {\mathbb {R}}^{p\times r}$ of full rank $r\le p$. However, it also holds that

21 $$\begin{aligned} P=ab'=\alpha \xi \beta ' \end{aligned}$$

for a non-singular matrix $\xi = (\beta '\alpha )^{-1}\Bigl (\exp (\delta \beta '\alpha )-I_r\Bigr )\in {\mathbb {R}}^{r\times r}$ and matrices $\alpha ,\beta \in {\mathbb {R}}^{p\times r}$, such that given weak conditions on the sampling timestep $\delta$ (see below), the following relations hold

$$\begin{aligned} {{\mathrm{rank}}}(P)&= {{\mathrm{rank}}}(\varPi ) = r \\ \text {sp}(\alpha )&=\text {sp}(a) \\ \text {sp}(\beta )&=\text {sp}(b), \end{aligned}$$

see Kessler and Rahbek (2001, 2004). Hence, for continuous time cointegrated processes, we can infer on the number of cointegration relations (${{\mathrm{rank}}}(\varPi )=r$) from discrete time observations, and also identify the subspaces spanned by the columns of $\alpha$ and $\beta$. Note however that due to the unidentified scaling $\xi$, we can only identify the subspaces, but not the parameters $\alpha ,\beta$ themselves. They are only unique up to a scaling ($\xi$), even though we have imposed the normalization (23) and thus uniquely identified *a* and *b*.

In the numerical part, we will refer to estimates of $\alpha$ and $\beta$, implicitly referring to the *discrete time* estimates. In terms of subspaces, there is no difference between the discrete and continuous time, but in order to interpret the *continuous time* $\varPi$ matrix, one must translate the discrete estimate to a continuous estimate using (19).

It is important to note that when working with continuous time models, one must be careful with regard to the relation (19) between discrete and continuous time and the sampling timestep $\delta$. Kessler and Rahbek (2004) refer to this issue as the *embedding problem*, and to ensure that the continuous time model is appropriate, one must check for $\exp (\delta \varPi )$ in (18) that it is non-singular, i.e., $|\exp (\delta \varPi )|\ne 0$, and that it has no negative eigenvalues. If this is the case and the underlying process is in fact cointegrated, the results above hold.

### Likelihood ratio test for ${{\mathrm{rank}}}(\varPi )=r$

Consider discrete observations $(\phi _{t_1},\ldots ,\phi _{t_N})$ from the continuous process (17) and denote by $H_r$ the hypothesis $H_r: {{\mathrm{rank}}}(\varPi )\le r$ for $r=0,\ldots ,p$. Then the set of hypotheses $H_0,\ldots ,H_r$ is nested,

$$\begin{aligned} H_0 \subseteq H_1 \subseteq \cdots \subseteq H_p, \end{aligned}$$

and $H_p$ correspond to the unrestricted model. The likelihood ratio test (LRT) that compare $H_r$ and $H_p$ is applied sequentially for $r=0,1,\ldots ,p-1$ and continued until $H_r$ against $H_p$ cannot be rejected, and thus determine the number of cointegrating relations for $\phi _t$. The LRT statistic is given by

22 $$\begin{aligned} -2\log Q(H_r|H_p) = \sum _{i=r+1}^p \hat{\lambda }_i, \end{aligned}$$

where $\hat{\lambda }_i$ are the solutions to the eigenvalue problem (49), see “Appendix 2”. The asymptotic distribution of (22) is non-standard and therefore it must be simulated. Here, to also improve on small-sample performance we perform *bootstrap* simulations as presented by Cavaliere et al. (2012), in order to determine critical values. Specifically, given the data $\{\phi _{t_n}\}_{n=1}^N$ bootstrap sequences $\{\phi _{t_n}^{*(m)}\}_{n=1}^N$ for $m=1,\ldots ,M$ are simulated and for each sequence the LRT statistic $\text {LRT}^{*(m)}$ is re-computed. The empirical quantiles of $\{\text {LRT}^{*(m)}\}_{m=1}^M$ are then used for testing. With *r* determined, $\hat{\beta }$ is given by the *r* eigenvectors corresponding to $\hat{\lambda }_i,i=1,\ldots ,r$ and the parameter estimates $\hat{\alpha },\hat{\mu },\hat{\varSigma }$ follow by ordinary least squares estimation as outlined in “Appendix 2”.

### Inference for $\alpha$ and $\beta$

Since we identify subspaces for $\alpha$ and $\beta$, then a normalization is necessary to identify the parameters uniquely. If $\hat{\beta }$ is known, then $\hat{\alpha }$ follows by OLS. Hence, if we impose a normalization on $\hat{\beta }$, we can identify all parameters. A common normalization, see Johansen (1996), is

$$\begin{aligned} \hat{\beta } = \tilde{\beta }(c'\tilde{\beta })^{-1}, \end{aligned}$$

where $c=(I_{r},0_{p-r\times r})'$ is a $p\times r$ matrix and $\tilde{\beta }$ is any version of the *r* eigenvectors corresponding to the *r* largest eigenvalues. This ensures that

23 $$\begin{aligned} {\hat{\beta }} = \begin{pmatrix} I_r \\ \tilde{\beta }_{p-r,r} \end{pmatrix}. \end{aligned}$$

Extending the idea of normalization to restrictions for $\alpha ,\beta$, we can impose such under the hypothesis $H_r$. Assume that ${{\mathrm{rank}}}(\varPi )=r$ and that the parameters $\alpha \in {\mathbb {R}}^{p\times r},\beta \in {\mathbb {R}}^{p\times r},\mu \in {\mathbb {R}}^p$ and $\varSigma \in {\mathbb {R}}^{p\times p}$ are all unrestricted within their corresponding subspaces, except for normalization (23). Possible hypotheses for $\alpha ,\beta$ are linear restrictions as given by

$$\begin{aligned} H_{\alpha }: \alpha&= A\psi \\ H_{\beta }: \beta&= B\xi , \end{aligned}$$

where $A\in {\mathbb {R}}^{p\times m}$, $\psi \in {\mathbb {R}}^{m\times r}$, $B\in {\mathbb {R}}^{p \times s}$, $\xi \in {\mathbb {R}}^{s \times r}$. The known matrices *A* and *B* represent the linear hypotheses and $\psi$ and $\xi$ are parameters to be estimated. It is also possible to combine the hypotheses for $\alpha$ and $\beta$ and we denote this $H_{\alpha ,\beta }$.

As an example, assume a system of 3 oscillators $\phi _t=(\phi _{1t},\phi _{2t},\phi _{3t})'$ with $r=1$. If we believe that $\phi _{3t}$ is independent of $\phi _{1t}$ and $\phi _{2t}$, we can specify the hypothesis

24 $$\begin{aligned} H_\alpha : A = \begin{pmatrix} 1 &{}\quad 0 \\ 0 &{}\quad 1 \\ 0 &{}\quad 0 \end{pmatrix}, \end{aligned}$$

such that

$$\begin{aligned} \varPi _A=\alpha _A\beta '=\begin{pmatrix}\psi _1 \\ \psi _2 \\ 0 \end{pmatrix} (\beta _1,\beta _2,\beta _3) = \begin{pmatrix} \psi _1\beta _1 &{} \quad \psi _1\beta _2 &{} \quad \psi _1\beta _3 \\ \psi _2\beta _1 &{} \quad \psi _2\beta _2 &{} \quad \psi _2\beta _3 \\ 0 &{} 0 &{} 0 \end{pmatrix}. \end{aligned}$$

This restriction imply that $\phi _{1t}$ and $\phi _{2t}$ do not contribute to the dynamics of $\phi _{3t}$, and hence that the latter is independent.

If we want to investigate a possible 1:1 coupling between $\phi _{1t}$ and $\phi _{2t}$, we can specify

25 $$\begin{aligned} H_\beta : B = \begin{pmatrix} 1\\ -1\\ 0 \end{pmatrix}, \end{aligned}$$

and obtain

$$\begin{aligned} \varPi _B=\alpha \beta '_B=\begin{pmatrix} \alpha _1\\ \alpha _2\\ \alpha _3 \end{pmatrix} (\eta ,-\eta ,0) = \begin{pmatrix} \alpha _1\eta &{} \quad -\alpha _1\eta &{} \quad 0 \\ \alpha _2\eta &{} \quad -\alpha _2\eta &{} \quad 0 \\ \alpha _3\eta &{} \quad -\alpha _3\eta &{} \quad 0 \\ \end{pmatrix}. \end{aligned}$$

Note however, that under $H_\beta$ the interaction between $\phi _{1t}$ and $\phi _{2t}$ also influence $\phi _{3t}$ if $\alpha _3\ne 0$. Hence, the system admits the relations $\phi _{1t}\leftrightarrow \phi _{2t}$, $\phi _{1t}\rightarrow \phi _{3t}$ and $\phi _{2t}\rightarrow \phi _{3t}$, where the restriction $\beta _3=0$ implies that the last two relations are uni-directional.

If we believe that $\phi _{1t}$ and $\phi _{2t}$ are bi-directionally coupled, $\phi _{1t}\leftrightarrow \phi _{2t}$, but $\phi _{3t}$ is independent and does not contribute to either $\phi _{1t}$ nor $\phi _{2t}$, we can phrase this hypothesis as a combination of (24) and (25). This leads to the restricted matrix

$$\begin{aligned} \varPi _{A,B}=\alpha _A\beta '_B=\begin{pmatrix} \psi _1 \\ \psi _2 \\ 0\end{pmatrix}(\eta ,-\eta ,0)= \begin{pmatrix} \psi _1\eta &{} \quad -\psi _1\eta &{} \quad 0 \\ \psi _2\eta &{} \quad -\psi _2\eta &{} \quad 0 \\ 0 &{} 0 &{} \quad 0 \end{pmatrix}. \end{aligned}$$

Other hypotheses, such as equal or proportional coupling strength or *l* : *n* coupling, can be specified by appropriately designing the matrices *A* and *B*. Thus, a broad variety of linear hypotheses on the parameter $\varPi =\alpha \beta '$ can be investigated, notably inference on the coupling directions and the effect of system disequilibrium on individual oscillators.

Evaluation of the hypotheses $H_\alpha ,H_\beta$, and $H_{\alpha ,\beta }$ all lead to similar likelihood ratio tests. To calculate the test statistic, solve again the eigenvalue problem (49) for the unrestricted model, and dependent on the restrictions *A* and/or *B* obtain eigenvalues $\lambda _i^*$ for the restricted model. The LRT statistic is then given by

26 $$\begin{aligned} -2\log Q\bigl (H_0|H_r \bigr ) = T\sum _{i=1}^r \log \left( \frac{1-\lambda _i^*}{1-\hat{\lambda }_i} \right) , \end{aligned}$$

where $H_0$ is a generic substitute for any of $H_\alpha ,H_\beta ,H_{\alpha ,\beta }$. Each of these statistics has an asymptotic $\chi ^2$ distribution with varying degrees of freedom (df),

$$\begin{aligned} -2\log Q\bigl (H_\alpha |H(p) \bigr )&\text { has } r(p-m) \text { df}\\ -2\log Q\bigl (H_\beta |H(p) \bigr )&\text { has } r(p-s) \text { df}\\ -2\log Q\bigl (H_{\alpha ,\beta }|H(p) \bigr )&\text { has } r(p-m)+r(p-s) \text { df}, \end{aligned}$$

where *m* and *s* are the column dimensions of the matrices *A* and *B*, respectively. This shows that once ${{\mathrm{rank}}}(\varPi )$ is determined, statistical inference for $\alpha$ and $\beta$ can be carried out, relatively straightforward. As for the rank determination, an alternative to the $\chi ^2$ approximation for inference on $\alpha$ and $\beta$ is to perform bootstrapping for the test (26), see Boswijk et al. (2016).

## Numerical simulations

### General setup

We perform a series of experiments with a system of $p=3$ linearly coupled Winfree oscillators such that $z_t\in {\mathbb {R}}^6$ and $f(\phi _t) = \alpha \beta '\phi _t$. Hence, for $i=1,2,3$, we have a DGP with

27 $$\begin{aligned} g(z_t)_i = f(\phi _t)_i = (\alpha \beta '\phi _t)_i=\alpha _i \sum _{j=1}^3 \beta _j\phi _{jt}. \end{aligned}$$

Since we examine simulations from the Winfree oscillator, our cointegration model will be misspecified since the amplitude is not deterministic and linear, but rather stochastic and fluctuating. However, since the amplitude of the Winfree oscillator has a relatively steady level (of course this also depends on the noise level), due to the squared multiplicative term in the amplitude process, we can approximate it as a constant. Hence we will do so in terms of analyzing the phase process as a cointegrating system. This also implies in terms of parameter estimation for the phase process, the estimate of the constant $\mu$ is a pseudo estimate of the $\kappa$ parameter for the amplitude process, and hence we will compare the estimates to the true value of $\kappa$.

For each experiment we simulate 1.000.000 iterations of the oscillator (10) using the Euler–Maruyama scheme with timestep $\widetilde{\varDelta }t = 0.0002$ and then subsample for $\varDelta t=0.1$, thus obtaining $N=2000$ (equidistant and discrete) observations of $z_{t}$ for $t\in [0,200)$. Subsampling every 5000th values diminishes the discretization error of the simulation scheme.

We use the same initial conditions,

$$\begin{aligned} z_0 = (1,0,0,1,-1,0)', \end{aligned}$$

and parameters

28 $$\begin{aligned} \begin{aligned} \kappa&= (0.75, 1, 1)' \\ \varSigma _\phi&= {{\mathrm{diag}}}(1,1,1)\\ \varSigma _\gamma&= {{\mathrm{diag}}}(0.1,0.1,0.1) \end{aligned} \end{aligned}$$

for all the experiments so that the only varying parameter is the coupling structure.

**Fig. 1:**
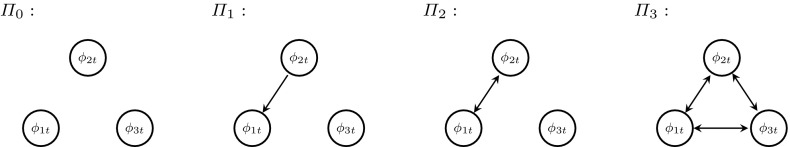
Graphical representation of the four systems, represented by the $\varPi _i, i=0,1,2,3$ matrix. The *arrows* define the direction of interaction, hence $\phi _{2t}\rightarrow \phi _{1t}$ implies that $\phi _{2t}$ is influencing $\phi _{1t}$ (uni-directional coupling), and $\phi _{2t}\leftrightarrow \phi _{1t}$ denotes bi-directional coupling, i.e. $\phi _{1t},\phi _{2t}$ influence eachother

Note that the $\kappa$ parameter for $\phi _{2t}$ is set equal to $\phi _{3t}$ to obtain similar simulated outcomes for some experiments to investigate whether we can distinguish between interaction and independence between these two. We set the cointegration parameters for each experiment individually to impose different coupling structures, and will refer to the relevant model by it’s $\varPi _k,k=0,1,2,3$ matrix, where *k* defines the model structure (see Fig. 1).

The discrete time model fitted to the data is specified as

29 $$\begin{aligned} \varDelta \phi _n = P\phi _{n-1}-\mu +\epsilon _n, \end{aligned}$$

where the estimate $\hat{P}$ is used to obtain $\hat{\varPi }$ through (20). The reported estimate for $\hat{\mu }$ is scaled by the timestep $\varDelta t$. Note that $\mu$ is not time-dependent and hence this model will fit a constant parameter $\mu$ to a varying quantity $\gamma _t$ and thus it is misspecified as mentioned above. Model (29) is estimated for all 4 systems of three oscillators and we report the parameters $\hat{\varPi }$ and $\hat{\mu }$ for each system. The latter is compared to $\kappa$, which is the level parameter of the $\gamma _t$ process.

In addition to a cointegration analysis we apply the *mean phase coherence measure*, see Mormann et al. (2000), bilaterally to the *wrapped* phases (i.e., $\phi _{it}\in [0,2\pi )$ for $i=1,2,3$)

30 $$\begin{aligned} R(\phi _{it},\phi _{jt}) = \biggl | \frac{1}{N} \sum _{n=1}^N e^{i(\phi _{i,t_n} - \phi _{j,t_n}) } \biggr |, \end{aligned}$$

as an initial measure of synchronization between the phases in the system. If $R\approx 1$ this implies synchronization ($R=1$ means that oscillators are perfectly phase locked). On the contrary, $R\approx 0$ implies that the distribution of phase differences is approximately uniformly distributed on the unit circle. Note that the mean phase coherence measure is symmetrical, like correlation, and therefore it cannot reveal uni-directional coupling. In order to determine the significance of the *R* measures, we bootstrapped critical values for the hypothesis $R=0$. Hence, these values are the same for all experiments and presented along with the measured *R* values. We compare the resulting value of *R* to the conclusion of the cointegration analysis.

**Fig. 2:**
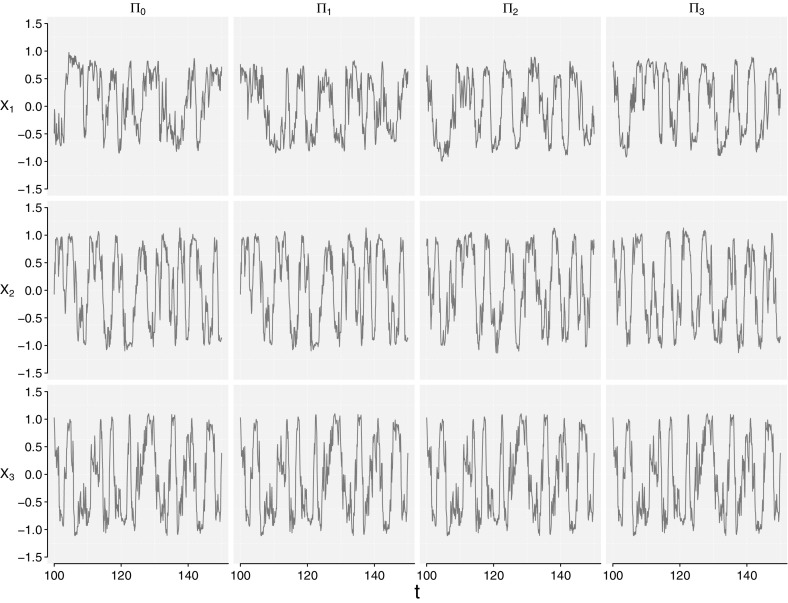
50 observations (x-coordinates only) from numerical simulation of the Winfree oscillator: the $\varPi _0$ column displays the independent model (31), the $\varPi _1$ column displays the uni-directional coupled model (32), the $\varPi _2$ column displays the bi-directional coupled model (33) and the $\varPi _3$ column displays the fully coupled model (34)

We use the same seed for all experiments so that the outcomes are fully comparable in terms of stochastic noise *dW*. First we run a simulation with uncoupled oscillators as a benchmark, and then continue with coupled systems as presented in Fig. 1. Figure 2 display the *x*-coordinates for $t\in [100,150]$ from a simulation of these four systems.

The data analysis is carried out using the free software package R (R Core Team 2015). The source code for simulation and bootstrapping procedures are written in C++ to decrease the runtime, utilizing the interface package Rcpp for R and linear algebra package RcppArmadillo for C++. The source code is available in the package cods as supplementary material.

### Independent oscillators

This experiment is used as a reference example. We set

31 $$\begin{aligned} \varPi _0 = \begin{pmatrix} 0 &{}\quad 0 &{}\quad 0 \\ 0 &{}\quad 0 &{}\quad 0 \\ 0 &{}\quad 0 &{}\quad 0 \end{pmatrix}, \end{aligned}$$

so ${{\mathrm{rank}}}(\varPi _0)=0$ and there is no interaction in the system.

**Fig. 3:**
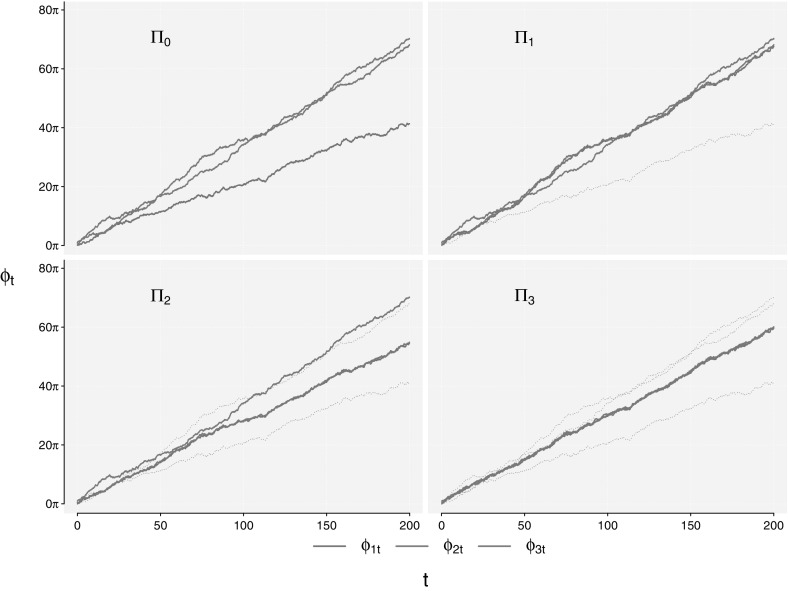
Unwrapped phase processes from numerical simulation of the Winfree oscillator: $\varPi _0$ displays the independent model (31), $\varPi _1$ displays the uni-directional coupled model (32), $\varPi _2$ displays the bi-directional coupled model (33) and $\varPi _3$ displays the fully coupled model (34). The *dotted lines* represent the corresponding phases from the independent model in $\varPi _0$

Simulating the model and unwrapping the phases, we obtain the top-left plot of Fig. 3.

Visual inspection of the plot could lead to the conclusion that $\phi _{2t}$ and $\phi _{3t}$ are coupled, however the mean phase coherence measure *R* for the phases indicates that this is not the case.

$$\begin{aligned} R(\phi _{1t},\phi _{2t})&= 0.025 \quad (0.170)\\ R(\phi _{1t},\phi _{3t})&= 0.073 \quad (0.168)\\ R(\phi _{2t},\phi _{3t})&= 0.078 \quad (0.176). \end{aligned}$$

The distribution of the mean phase coherence measure is unknown, but can be approximated by bootstrapping for $H_0: R=0$, that is for no synchronization present. 1000 bootstrap samples yield the reported 5% critical values in parentheses above $\approx$0.17, thus the mean phase coherence measure suggest no synchronization present, which is the case.

Performing now a rank test for the rank of $\varPi _0$ in the system, we obtain the first part of Table 1.

**Table 1:** Rank tests for models $\varPi _i, i=0,1,2,3$ with the selected models indicated in bold

Model	$H_r$	Test values	*p* value
$\varPi _0$	$\mathbf {r=0}$	**16**.**47**	**0**.**663**
	$r\le 1$	3.94	0.753
	$r\le 2$	0.05	0.812
$\varPi _1$	$r=0$	118.39	0.000
	$\mathbf {r\le 1}$	**4**.**30**	**0**.**568**
	$r\le 2$	0.00	0.958
$\varPi _2$	$r=0$	104.48	0.000
	$\mathbf {r\le 1}$	**3**.**84**	**0**.**707**
	$r\le 2$	0.03	0.843
$\varPi _3$	$r=0$	157.81	0.000
	$r\le 1$	63.82	0.000
	$\mathbf {r\le 2}$	**0**.**00**	**0**.**947**

The test values are given by Eq. (22) and *p* values are determined by bootstrapping

The test does not reject the hypothesis $H_r: r=0$, thus suggesting that there is no cointegration present in the system. This in turn implies that the oscillators are independent in terms of synchronization, in accordance with the DGP for $\varPi _0$, and with the mean phase coherence measure.

### Uni-directional coupling

In this experiment we analyze a system with a uni-directional coupling. Let

32 $$\begin{aligned} \begin{aligned} \alpha&= (-0.5,0,0)'\\ \beta&= (1,-1,0)' \end{aligned} \end{aligned}$$

such that ${{\mathrm{rank}}}(\varPi _1)={{\mathrm{rank}}}(\alpha \beta ')=1$, and we have the stationary relation $\phi _{1t}-\phi _{2t}$. Since $\alpha _2=\alpha _3=0$, then $\phi _{2t}$ and $\phi _{3t}$ are acting independently, whereas $\phi _{1t}$ is influenced by $\phi _{2t}$. Hence, the only coupling is $\phi _{2t}\rightarrow \phi _{1t}$.

The unwrapped phases for the simulation of model $\varPi _1$ are seen in the top-right of Fig. 3. The dashed lines indicate the independent phases from the top-left of Fig. 3, and we see that phases $\phi _{2t},\phi _{3t}$ are equal to their independent versions, whereas we now clearly find that $\phi _{1t}$ is attracted towards $\phi _{2t}$ due to the coupling structure in the system.

Examining the mean phase coherence in Eq. (30) for the system (note that $R(\phi _{2t},\phi _{3t})$ is equal to the value in the previous section),

$$\begin{aligned} R(\phi _{1t},\phi _{2t})&= 0.321 \quad {(0.170)}\\ R(\phi _{1t},\phi _{3t})&= 0.049 \quad {(0.168)} \\ R(\phi _{2t},\phi _{3t})&= 0.078 \quad {(0.176)}, \end{aligned}$$

we find indications of some synchronization between the phases $\phi _{1t}$ and $\phi _{2t}$ in the system compared to $R(\phi _{1t},\phi _{2t})$ in the independent model. The value is significant on a 5% level as seen by the reported critical values, whereas for $R(\phi _{1t},\phi _{3t})$ and $R(\phi _{2t},\phi _{3t})$ the reported values are not. However, the mean phase coherence measure does not recognize the uni-directional coupling as is the case here. Thus, it cannot distinguish between $\phi _{1t}\rightarrow \phi _{2t}$, $\phi _{1t}\leftarrow \phi _{2t}$ and $\phi _{1t}\leftrightarrow \phi _{2t}$.

Results from the rank test are in the second part of Table 1. Here we see that $r={{\mathrm{rank}}}(\varPi _1)=0$ is clearly rejected, whereas $r=1$ cannot be rejected with a *p* value of 0.568. This indicates the presence of a single cointegration relation, in accordance with the construction of the model.

Fitting the model with $r=1$, we obtain the unrestricted MLE regression estimates in Table 2. The cointegration relations are close to their true values (approximately within 1 standard error), and both $\alpha _2$ and $\alpha _3$ are statistically insignificant. Moreover, the estimates of $\beta$ suggests a 1:1 coupling between $\phi _1$ and $\phi _2$.

**Table 2:** Fitted model $\varPi _1$

Parameter	True value	Unrestricted estimates			Restricted $\alpha$ and $\beta$		
		Estimate	SE	*p* value	Estimate	SE	*p* value
$\alpha _1$	$-$0.5	$-$0.527	0.049	<0.001	$-$0.514	0.048	<0.001
$\alpha _2$	0	$-$0.050	0.049	0.307	0		
$\alpha _3$	0	0.059	0.048	0.223	0		
$\beta _1$	1	1			1		
$\beta _2$	$-$1	$-$0.981			$-$1		
$\beta _3$	0	$-$0.016			0		
$\kappa _1$	0.75	0.765	0.076	<0.001	0.638	0.081	<0.001
$\kappa _2$	1	1.035	0.075	<0.001	1.063	0.080	<0.001
$\kappa _3$	1	1.119	0.074	<0.001	1.086	0.080	<0.001

Therefore, we perform a likelihood test for reducing the unrestricted model, with restrictions for both $\alpha$ and $\beta$

$$\begin{aligned} H_{\alpha ,\beta }: \alpha&= A\psi ,\quad \text { with }A =(1,0,0)'\\ \beta&= B\xi ,\quad \text { with }B = (1, -1, 0)', \end{aligned}$$

so that *A* fix $\alpha _2=\alpha _3=0$ and *B* restricts to a 1:1 coupling. This yields the test statistic 3.617 which is $\chi ^2$ distributed with 4 degrees of freedom and hence implies a *p* value of 0.460. Thus, we recover the true uni-directional coupling structure of the simulated phases. The fitted model is presented in the right of Table 2.

The conclusion is that we have successfully identified the coupling structure of uni-directional coupled phases in a three dimensional system, with two independent phases, and one dependent. Since $\phi _{3t}$ is completely independent of $\phi _{1t}$ and $\phi _{2t}$ and $r=1$ we can discard $\phi _{3t}$ when interpreting the cointegration in the system. Then we can interpret the cointegration parameter $\alpha$ as the coupling strength and $\beta$ as the coupling scheme, here 1:1. If we had analyzed different data, with a $\beta$ estimate close to $\hat{\beta }=(1,-n,0)'$, we could then identify a *n*:1 coupling between $\phi _{1t}$ and $\phi _{2t}$. This can be seen from the fact that in this case $\alpha _k(\phi _{1t}-n\phi _{2t})$ would be a stationary relation, and thus $\phi _{2t}$ would rotate $\approx$ *n* times slower than $\phi _{1t}$.

### A bi-directional coupling with one independent oscillator

We now look at a system with

33 $$\begin{aligned} \begin{aligned} \alpha&= (-0.5,0.5,0)'\\ \beta&= (1,-1,0)'. \end{aligned} \end{aligned}$$

Hence, ${{\mathrm{rank}}}(\varPi _2) = 1$ and again we have 1 stationary relation $\phi _{1t}-\phi _{2t}$, but now with only $\phi _{3t}$ independent, and a bidirectional coupling $\phi _{1t}\leftrightarrow \phi _{2t}$.

Simulating the $\varPi _2$-model we obtain the bottom-left of Fig. 3. We have included the dashed lines again, as references for the independent system. If we contrast the bottom-left of Fig. 3 with the top-right of Fig. 3, we now find that $\phi _{1t}$ and $\phi _{2t}$ are attracting each other, and hence they are both different from their independent versions. Since $|\alpha _1|=|\alpha _2|$, their coupling strength is equal, and the coupled phases lies roughly in the middle between the independent ones. If we look at the mean phase coherence measure for the pairwise interactions,

$$\begin{aligned} R(\phi _{1t},\phi _{2t})&= 0.590 \quad (0.170) \\ R(\phi _{1t},\phi _{3t})&= 0.144 \quad (0.168)\\ R(\phi _{2t},\phi _{3t})&= 0.126 \quad (0.176), \end{aligned}$$

we find relatively strong evidence of a coupling between the phases $\phi _{1t}$ and $\phi _{2t}$, the value is higher than in the uni-directional case and it is (again) significant given the bootstrapped critical values. However, again we cannot distinguish between types of coupling structures.

Performing a rank test for cointegration in the system with $\varPi _2$, we see in the third part of Table 1 that $H_r:r=0$ is clearly rejected, and we find that the rank of $\varPi _2$ is estimated to 1 with a *p* value of 0.707. Hence, we recover the correct dimension of the column space of $\beta$, and fitting a model with $r=1$ yields the parameters in the left of Table 3.

**Table 3:** Fitted model $\varPi _2$

Parameter	True value	Unrestricted estimates			Restricted $\alpha$ and $\beta$			Restricted $\alpha$ and $\beta$ with $A^*$		
		Estimate	SE	*p* value	Estimate	SE	*p* value	Estimate	SE	*p* value
$\alpha _1$	$-$0.5	$-$0.530	0.071	<0.001	$-$0.506	0.069	<0.001	$-$0.475	0.069	<0.001
$\alpha _2$	0.5	0.450	0.069	<0.001	0.443	0.067	<0.001	0.475	0.067	<0.001
$\alpha _3$	0	0.087	0.070	0.214	0			0		
$\beta _1$	1	1			1			1		
$\beta _2$	$-$1	$-$0.970			$-$1			$-$1		
$\beta _3$	0	$-$0.022			0			0		
$\kappa _1$	0.75	0.754	0.072	<0.001	0.646	0.076	<0.001	0.660	0.076	<0.001
$\kappa _2$	1	0.943	0.070	<0.001	1.040	0.074	<0.001	1.053	0.074	<0.001
$\kappa _3$	1	1.103	0.071	<0.001	1.086	0.075	<0.001	1.086	0.075	<0.001

The only insignificant parameter for the model is $\alpha _3$, which is in accordance with the construction of the $\varPi _2$ model. Specifying the hypothesis

$$\begin{aligned} H_{\alpha ,\beta }: \alpha&= A\psi ,\quad \text { with }A = \begin{pmatrix} 1 &{}\quad 0 \\ 0 &{}\quad 1 \\ 0 &{}\quad 0 \end{pmatrix}\\ \beta&= B\xi ,\quad \text { with }B = (1, -1, 0)', \end{aligned}$$

and performing a likelihood ratio test for the reduction yields a test statistic of 3.340, which follows a $\chi ^2$ with 3 degrees of freedom, and result in a *p* value of 0.342. The fitted model is given in the middle of Table 3. If we instead of $H_{\alpha ,\beta }$ specify

$$\begin{aligned} H_{\alpha ,\beta }^*: \alpha&= A\psi ,\quad \text { with }A =(1,-1,0)' \\ \beta&= B\xi ,\quad \text { with }B = (1, -1, 0)', \end{aligned}$$

implying that $\alpha _1 = -\alpha _2$, we obtain a test statistic of 3.880, with 4 degrees of freedom, and a *p* value of 0.423. Thus, we can also restrict the model to one where the coupling strengths are equal in magnitude. The fitted model is presented in the right part of Table 3.

Summing up, in a system of bi-directional coupled oscillators plus one independent, we can identify the correct coupling between them, including identifying the proportionally equal coupling strength between the coupled phases. Again we identify $r=1$, and hence we can interpret the cointegration parameters as before, hence $\alpha$ is the coupling strength, and $\beta$ the interaction, again 1:1 coupling.

### Fully coupled system

We specify a system with full interaction between all phases.

34 $$\begin{aligned} \begin{aligned} \alpha&= \begin{pmatrix} -0.50 &{}\quad 0.25 \\ 0.25 &{}\quad -0.50 \\ 0.25 &{}\quad 0.25 \end{pmatrix}\\ \beta&= \begin{pmatrix} 1 &{}\quad 0 \\ 0 &{}\quad 1 \\ -1 &{}\quad -1 \\ \end{pmatrix}. \end{aligned} \end{aligned}$$

The $\alpha$ and $\beta$ matrix are chosen, such that

$$\begin{aligned} \varPi _3 = \alpha \beta ' = \begin{pmatrix} -0.50 &{}\quad 0.25 &{}\quad 0.25 \\ 0.25 &{}\quad -0.50 &{}\quad 0.25 \\ 0.25 &{}\quad 0.25 &{}\quad -0.50 \end{pmatrix} \end{aligned}$$

inspired by the simplistic linearization of the Kuramoto model, as presented in Eq. (14). Note that ${{\mathrm{rank}}}(\varPi _3)=2$.

The simulated phases are shown in the bottom-right of Fig. 3. Comparing to the dashed (independent) versions, we now find that all phases are different from their independent versions. It appears as if $\phi _{2t},\phi _{3t}$ dominate the system, since $\phi _{1t}$ is attracted closer to their independent versions than otherwise, but it is also a two-against one ($\kappa _2=\kappa _3\ne \kappa _1$) scheme, and we roughly observe that $\phi _{1t}$ is attracted 2/3 towards $\phi _{2t},\phi _{3t}$, whereas $\phi _{2t},\phi _{3t}$ are attracted 1 / 3 towards $\phi _{1t}$. So by the construction of the system, this behavior seems natural. We find that the mean phase coherence measure

$$\begin{aligned} R(\phi _{1t},\phi _{2t})&= 0.487 \quad (0.170) \\ R(\phi _{1t},\phi _{3t})&= 0.574 \quad (0.168) \\ R(\phi _{2t},\phi _{3t})&= 0.488 \quad (0.176), \end{aligned}$$

indicates bilateral synchronization for all phases, and all values are significant. The rank test also gives clear evidence of cointegration and we identify $r=2$, as seen in the bottom part of Table 1, where both the hypotheses $r=0$ and $r=1$ are rejected. Fitting a model with $r=2$ yields the left half of Table 4.

**Table 4:** Fitted model $\varPi _3$

Parameter	True value	Unrestricted estimates			Restricted $\alpha$ and $\beta$		
		Estimate	SE	*p* value	Estimate	SE	*p* value
$\alpha _{11}$	$-$0.50	$-$0.584	0.075	<0.001	$-$0.569	0.075	<0.001
$\alpha _{21}$	0.25	0.232	0.073	<0.001	0.241	0.072	<0.001
$\alpha _{31}$	0.25	0.326	0.072	<0.001	0.328	0.072	<0.001
$\alpha _{12}$	0.25	0.223	0.067	<0.001	0.224	0.067	<0.001
$\alpha _{22}$	$-$0.50	$-$0.423	0.064	<0.001	$-$0.423	0.064	<0.001
$\alpha _{32}$	0.25	0.201	0.064	<0.001	0.199	0.064	<0.001
$\beta _{11}$	1	1			1		
$\beta _{21}$	0	0			0		
$\beta _{31}$	$-$1	$-$0.997			$-$1		
$\beta _{12}$	0	0			0		
$\beta _{22}$	1	1			1		
$\beta _{32}$	$-$1	$-$0.999			$-$1		
$\kappa _1$	0.75	0.712	0.076	<0.001	0.607	0.083	<0.001
$\kappa _2$	1	1.054	0.074	<0.001	1.061	0.080	<0.001
$\kappa _2$	1	1.023	0.073	<0.001	1.130	0.080	<0.001

The estimated $\kappa$’s are close to their respective values, whereas some of the $\alpha$ parameters deviate (more than 1 standard error) from their true values. If we inspect the estimated $\hat{\varPi }_3$

$$\begin{aligned} \hat{\varPi }_3 = \begin{pmatrix} -0.611 &{}\quad 0.231 &{}\quad 0.378 \\ 0.241 &{}\quad -0.437 &{}\quad 0.196 \\ 0.343 &{}\quad 0.207 &{}\quad -0.549 \\ \end{pmatrix} \end{aligned}$$

and compare with the true $\varPi _3$ it looks better. The row sums are close to zero as they should be, and the signs are correct. The proportional coupling strengths are off though, especially between $\phi _{1t},\phi _{3t}$, but it seems that $\varPi _3$ is relatively well estimated considering the identification issues. Recall that we can determine the subspaces $\text {sp}(\alpha )$ and $\text {sp}(\beta )$ for continuous time cointegration models, see Kessler and Rahbek (2004), but that we have problems regarding the scaling of $\varPi$ (see Sect. 3.3).

Inspired by the fitted values, we restrict both matrices $\alpha$ and $\beta$

$$\begin{aligned} H_{\alpha ,\beta }: \alpha&= A\psi , \text { with }A = \begin{pmatrix} -0.50 &{}\quad 0.25 \\ 0.25 &{}\quad -0.50 \\ 0.25 &{}\quad 0.25 \end{pmatrix}\\ \beta&= B\xi , \text { with }B = \begin{pmatrix} 1 &{}\quad 0 \\ 0 &{}\quad 1 \\ -1 &{}\quad -1 \\ \end{pmatrix} \end{aligned}$$

we find that the test statistic is 1.73, $\chi ^2$ distributed with 4 degrees of freedom, and thus a *p* value of 0.785. Hence, we can reduce the model to one with restrictions that generates the true structure of $\varPi$. The estimated model parameters are presented in Table 4, and the corresponding $\hat{\varPi }$ is

$$\begin{aligned} \hat{\varPi }_3^* = \begin{pmatrix} -0.595 &{}\quad 0.232 &{}\quad 0.363 \\ 0.250 &{}\quad -0.438 &{}\quad 0.188 \\ 0.345 &{}\quad 0.205 &{}\quad -0.550 \end{pmatrix}. \end{aligned}$$

Concluding on the fully coupled system, we find that we can correctly identify the dimension of the cointegration relations. We can also determine the coupling structure as given by the parameters $\alpha$ and $\beta$. However, interpretation in this experiment is more informative in terms of $\hat{\varPi }$, since with $r\ge 2$, the interpretation of cointegration parameters is not as intuitive as in the case of $r=1$. We obtain a $\varPi$ estimate that is reminiscent of the true matrix, with the true directions of the coupling, and strengths somewhat close to the actual values. Thus, we can interpret the system as fully coupled, in a simplistic (linear) Kuramoto type model.

### Strength of coupling and identification of interaction

In this section, we compare the mean phase coherence measure to the cointegration analysis with respect to interactions in the system. More specifically, we look at how strong the coupling constants in $\varPi$ must be in order for the two methods to conclude correctly on interaction in the system. We reuse the parameter settings (34) from the fully coupled experiment, but use a scaled $\varPi$ matrix $\varPi \rightarrow \varepsilon \varPi$, for $\varepsilon \in [0,1]$, where $\varepsilon$ controls the coupling strength. The higher $\varepsilon$ is, the stronger the coupling, and hence the attraction between phases. Note that $\varepsilon =0$ corresponds to the model $\varPi _0$ and $\varepsilon =1$ corresponds to $\varPi _3$. The *p* values are calculated using bootstrapping as presented by Cavaliere et al. (2012) to obtain an estimate of the asymptotic distribution of the trace test statistics.

The aim is to investigate the rank test for varying $\varepsilon$ compared to identification of interaction in the system using the mean phase coherence measure. Since low values of $\varepsilon$ implies weak interaction, the expectation is that both methods will produce doubtful results in a low value regime. From the previous experiment on the fully coupled oscillators, the mean phase coherence measure produced low values on identifying the interaction of the system, hence we expect that the rank test will outperform for low values of $\varepsilon$.

**Fig. 4:**
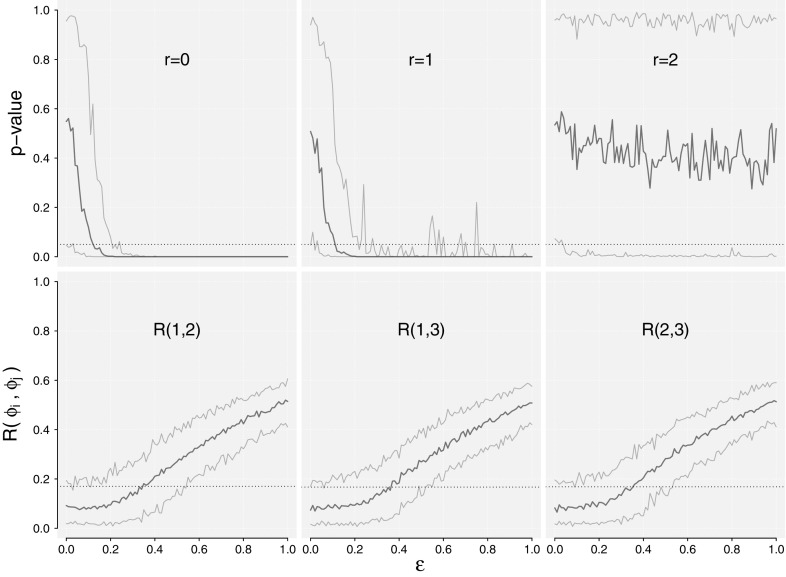
Identification of interaction for varying coupling strengths $\epsilon$ for the model $\varepsilon \varPi _3$. *Top row* are hypotheses $H_r: r=0,1,2$ using the trace test. The *orange bounds* indicates empirical quantiles ranging from 2.5 to $97.5\%$ and the *blue lines* represents the median. The *bottom row* are mean phase coherence measures again with empirical quantiles illustrated. *Dashed lines* in the *top row* show the 5% confidence levels. *Dashed lines* in the *bottom row* show the 95% quantile for $H_0: R(i,j)=0$ at $\varepsilon =0$, found by bootstrapping (color figure online)

The experimental setup is 100 repetitions for each value of $\varepsilon$, and in each repetition perform 500 bootstrap samples to estimate the *p* value for the hypotheses $H_r: r=0,1,2$. Figure 4 presents the median *p* values for the rank test and median mean phase coherence measures against $\varepsilon$. The top row of the figure shows the *p* values for $H_r: r=0,1,2$ respectively, and the bottom row shows the mean phase coherence (*R*) measures for pairs of $\phi _{1t},\phi _{2t}$ and $\phi _{3t}$. The dotted lines indicate the $p=0.05$ value, under which we reject the hypothesis. For the mean phase coherence measure, the 95% significance level for the hypothesis $R=0$ has been determined numerically using bootstrapping and is indicated by the dotted lines. If the *R*-measure falls below this line, independence cannot be rejected.

Seen in the top row of Fig. 4, at least half the simulations reject $H_r: r=0$ for $\varepsilon > 0.12$, and at least half the simulations reject $H_r: r=1$ for $\varepsilon >0.11$. The test does not reject $H_r: r=2$ for around 88% of the simulated samples for any values of $\varepsilon$. Thus, for $\varepsilon >0.11$, we can conclude that there is interaction present in the system, and in most of the simulations we also recognize the true ${{\mathrm{rank}}}(\varPi )=2$.

If we turn to the bottom row of Fig. 4, where the mean phase coherence measures are shown, we find that half the simulations does not reject the hypothesis $R=0$ for $\varepsilon <0.34,0.36$ and 0.35, respectively, for $R(\phi _{1t},\phi _{2t})$,$R(\phi _{1t},\phi _{3t})$ and $R(\phi _{2t},\phi _{3t})$, thus clearly indicating an inferior detection of interaction for small values of $\varepsilon$ equivalent to weak couplings.

Concluding on this experiment, we find that the rank test detects interaction in the system already at relatively weak coupling strengths. In contrast to this, the coupling must be significantly stronger for a sound conclusion on interaction in the system when using mean phase coherence as a measure of interaction. Furthermore, when detecting interaction in the system, the rank test is also very capable of identifying the true rank of the system, despite a misspecified model. Higher sample sizes will of course improve the inference results.

### Consistency of the rank estimation

To investigate the consistency of the cointegration algorithm, we performed an experiment with 1000 repetitions of simulations for Winfree oscillators, the uni-directional coupling, the bi-directional and the fully coupled systems, respectively, and evaluating the rank test, using the same setup as in Sect. 4.1. Table 5 present the percentages of conclusions regarding hypotheses $H_r: r=0, r\le 1,2,3$, for each model. Comparing with critical values at a $5\%$ level, obtained by bootstrapping, see Cavaliere et al. (2012), we find that comparing the percentage of simulations where the test correctly identifies the cointegration rank of 1 for uni- and bi-directional coupling are 76.8 and $69.8\%$, respectively, at a 5% significance level. For the fully coupled system the percentage is $85.5\%$, and for an independent system the percentage is $96.2\%$.

**Table 5:** Percentage of conclusions on ${{\mathrm{rank}}}(\varPi )$, at a 5% significance level for a sample size of 2000

Model	$r=0$	$r\le 1$	$r\le 2$	$r\le 3$
Independent (%)	**96.2**	2.2	1.3	0.3
Uni-directional (%)	1.7	**76.8**	19.0	2.5
Bi-directional (%)	2.4	**69.8**	24.7	3.1
Fully coupled (%)	0.0	1.3	**85.5**	13.2

Note that the conclusion $r\le 3$ means that $\varPi$ is of full rank and therefore invertible, hence $\beta =I_3$. Correct conclusions in bold

**Table 6:** Percentage of conclusions on interaction indicated by the rank test and the mean phase coherence measures, at a 5% significance level for a sample size of 2000

Model	Rank test	$R(\phi _{1t},\phi _{2t})$	$R(\phi _{1t},\phi _{3t})$	$R(\phi _{2t},\phi _{3t})$
Independent (%)	3.8	4.7	4.4	5.7
Uni-directional (%)	98.3	99.8	5.6	4.4
Bi-directional (%)	97.6	100.0	7.2	7.0
Fully coupled (%)	100	100.0	100	100.0

These results show that identification of interaction in a system of coupled oscillators is quite precise, and the rank is underestimated in $\le$2.5% of the simulations for any model. In the case of independent or full interaction, the method is very good, whereas for systems with directed interaction, or interaction among some oscillators the frequency of overestimating the rank is $\approx$20–25%. This discrepancy seems intuitively correct, since for the latter systems the true model is a subset of the model of higher rank. As before higher sample sizes will of course improve the inference results.

In Table 6 we compare, in percentages, the conclusions on interaction in the systems, for each model. The values for the rank test presented here, are the summed values from Table 5 for $r\ne 0$. We find that both methods are very adept in identifying interaction in these systems. The results, however, should be held against the previous section, where the rank test outperformed the mean phase coherence measure for weak coupling strength. Also noting the fact, that the mean phase coherence measure cannot account for uni-directional coupling, our overall conclusion is that in terms of identifying interaction in the system, the methods seem to perform equally well for stronger coupling, whereas in explaining the system architecture, a cointegration analysis leaves us with more information on how the network is constructed.

## Analysis of EEG data

**Fig. 5:**
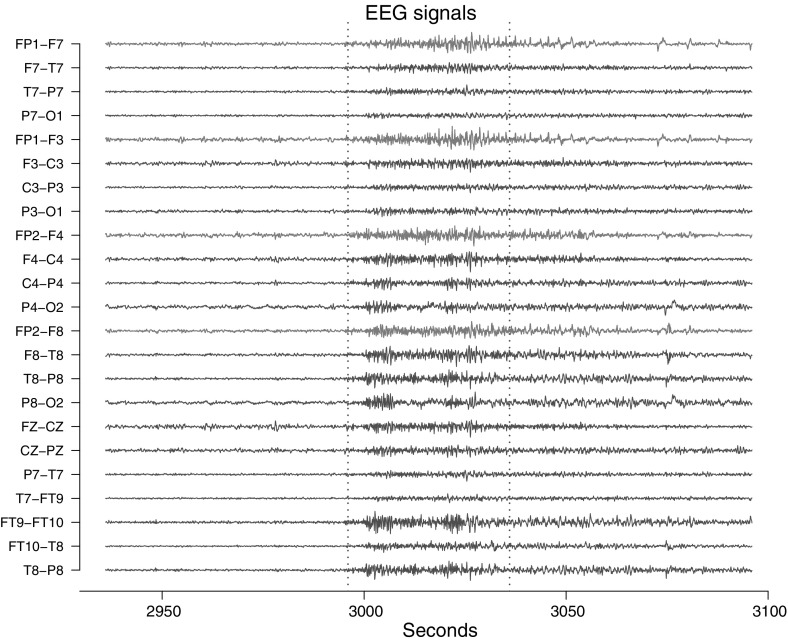
EEG recording leading op to a seizure and afterwards for a 11 year old female subject. The interval [2996;3036] s, as indicated by the *vertical red dashed lines*, is defined by Shoeb (2009) as a seizure. We analyze the four *blue signals*, FP1-F7, FP1-F3, FP2-F4 and FP2-F8 (color figure online)

Electroencephalography (EEG) signals are recordings from electrodes distributed on the scalp of subjects. The recorded brainwave patterns are, among others, used for diagnosing sleep disorders, coma or epilepsy. A study on 22 subjects experiencing epileptic seizures from the Children’s Hospital Boston is presented by Shoeb (2009) with the aim of detecting seizures based on multiple hours of recordings for each individual. Figure 5 displays an EEG recording of a single subject during a period that include a seizure identified by Shoeb (2009) between 2996 and 3036 s. The seizure is marked by two red dashed lines in Fig. 5. The labels for the signals refer to the individual electrodes on the scalp. We analyze the four signals FP1-F7, FP1-F3, FP2-F4 and FP2-F8, where FP refer to the frontal lobes and F refer to a row of electrodes placed behind these. Even numbered electrodes are on the right side and odd numbered electrodes are on the left side. Smaller (larger) numberings imply that the electrode is placed closer to (further from) the center of the scalp. Hence FP1-F7, FP1-F3 are measurements from the left side, with F3 placed closer to the center than F7, and likewise for right side signals FP2-F4 and FP2-F8. The electrodes for these four signals mirror each other on the left/right side of the scalp. We analyze the seizure period of 40 s and the 40 s leading up to the seizure, i.e. we analyze the two intervals [2956; 2996] and [2996; 3036] respectively, and refer to these as *prior to seizure* and *during seizure*. With a sample frequency of 256 measurements each second there are a total of 10,240 measurements for each of the four signals during the 40 s intervals. For more details on the data, see Shoeb (2009). The objective is to compare two fitted cointegration models with interaction as in Eq. (8) for each period:

$$\begin{aligned} d\phi _t = \alpha \beta ' \begin{pmatrix} \phi _{t,\text {FP1-F3}}\\ \phi _{t,\text {FP1-F7}}\\ \phi _{t,\text {FP2-F4}}\\ \phi _{t,\text {FP2-F8}} \end{pmatrix}dt+\mu dt+\varSigma dW_t, \end{aligned}$$

discretely observed for $t=1,\ldots ,10{,}240$ in each of the two intervals.

**Fig. 6:**
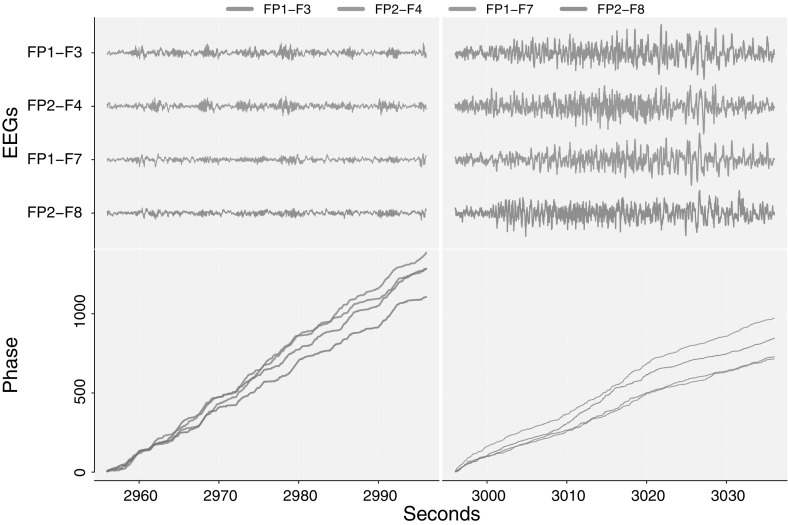
FP1-F7, FP1-F3, FP2-F4 EEG signals and estimated phase processes for a 11 year old female subject. *Top left* EEG signals prior to a seizure. *Top right* EEG signals during a seizure. *Bottom left* estimated phase processes prior to a seizure. *Bottom right* estimated phase processes during a seizure

The phase processes of the four signals are estimated using the Hilbert transform (see Sect. 2.3). Figure 6 shows the four signals in the two periods and their corresponding estimated unwrapped phase processes. Hence the offsets are in $[0,2\pi )$ for the individual phase processes in each period. If we had not split the measurements at 2996 s, the phases in the bottom right of Fig. 6 would be continuations of the phases in the bottom left. A visual inspection of Fig. 6 shows that when transitioning to the seizure period, the phases change to a slower pace (the slopes decrease). Also, prior to the seizure all four phases are closer with no clear distinction between right side and left side phases. During the seizure, the phases split in two groups: right and left side respectively.

This indicates that the model regime changes when transitioning into the seizure period. Table 7 shows the mean phase coherence measures bilaterally for the 4 phase processes and the average of these. Comparing the columns we find no clear indication of a change in the phase regime when transitioning into the seizure period based on this measure, the average change is only 7.5%. However, the measure does indicate interaction in the system among all phases.

**Table 7:** Mean phase coherence measures for EEG phases prior to and during the seizure

	Prior to seizure	During seizure
$R_{(\text {FP1-F3; FP2-F4)}}$	0.480	0.542
$R_{(\text {FP1-F3; FP1-F7)}}$	0.535	0.644
$R_{(\text {FP1-F3; FP2-F8)}}$	0.295	0.184
$R_{(\text {FP2-F4; FP1-F7)}}$	0.321	0.350
$R_{(\text {FP2-F4; FP2-F8)}}$	0.486	0.342
$R_{(\text {FP1-F7; FP2-F8)}}$	0.525	0.379
Average	0.440	0.407

Table 8 displays the results of a rank test procedure for the system of the four EEG phase processes.

**Table 8:** Rank tests for EEG phases in the bottom of Fig. 6

$H_r$	Prior to seizure		During seizure	
	Test values	*p* value	Test values	*p* value
$r=0$	105.87	0.000	1132.64	0.000
$r\le 1$	42.82	0.000	41.68	0.008
$\mathbf {r\le 2}$	**9**.**98**	**0**.**053**	**7**.**19**	**0**.**618**
$r\le 3$	0.46	0.439	0.72	0.786

The rank is determined to $r=2$ in both periods, although the conclusion is far stronger during the seizure. The significance of the statistics are found using 5000 bootstrap samples prior to the seizure due the border limit case of around 5%, during the seizure the *p* value is determined from 2000 bootstrap samples

In accordance with the indications from the mean phase coherence measure, the conclusion is a clear presence of cointegration during both periods. Prior to the seizure the rank test of $r\le 2$ is close to the usual 5% significance level, hence the *p* value here is determined using 5000 bootstrap samples, in contrast to the 2000 bootstrap samples used in the other interval, as the conclusion here is quite clear with a *p* value $\approx$0.62. In both cases we choose the rank $r=2$ for the system.

The fitted models are presented in Table 9 with the model fit prior to the seizure on the left side and the fit during the seizure on the right side. If we first note the estimated $\mu _i$’s, these are larger during the seizure and significantly so for FP1-F3 and FP2-F4, implying that these phase processes exhibit significantly higher intrinsic linear trends during the seizure. On the other hand, directly interpreting the cointegration parameters is not clear. Recall that these parameters specify subspaces, in this case within ${\mathbb {R}}^4$. We therefore look at the estimated $\hat{\varPi }$ matrices in Table 10 to compare the models for each period.

**Table 9:** Fitted model for EEG phases F7-T7, T7-P7 and FP1-F7

Parameter	Prior to seizure			During seizure		
	Estimate	SE	*p* value	Estimate	SE	*p* value
$\alpha _{\text {FP1-F3},1}$	$-$0.100	0.018	<0.001	$-$0.462	0.028	<0.001
$\alpha _{\text {FP1-F7},1}$	$-$0.002	0.019	0.930	$-$0.308	0.032	<0.001
$\alpha _{\text {FP2-F4},1}$	$-$0.035	0.017	0.044	$-$0.722	0.035	<0.001
$\alpha _{\text {FP2-F8},1}$	$-$0.115	0.030	<0.001	$-$0.648	0.042	<0.001
$\alpha _{\text {FP1-F3},2}$	$-$0.117	0.016	<0.001	0.041	0.033	0.212
$\alpha _{\text {FP1-F7},2}$	$-$0.024	0.016	0.147	0.071	0.037	0.057
$\alpha _{\text {FP2-F4},2}$	$-$0.026	0.015	0.084	0.173	0.041	<0.001
$\alpha _{\text {FP2-F8},2}$	$-$0.049	0.026	0.063	0.468	0.049	<0.001
$\beta _{\text {FP1-F3},1}$	1			1		
$\beta _{\text {FP1-F7},1}$	0			0		
$\beta _{\text {FP2-F4},1}$	$-$3.424			$-$0.036		
$\beta _{\text {FP2-F8},1}$	2.610			$-$0.573		
$\beta _{\text {FP1-F3},2}$	0			0		
$\beta _{\text {FP1-F7},2}$	1			1		
$\beta _{\text {FP2-F4},2}$	2.486			$-$0.840		
$\beta _{\text {FP2-F8},2}$	$-$3.631			0.188		
$\mu _\text {FP1-F3}$	25.210	2.162	<0.001	39.647	1.307	<0.001
$\mu _\text {FP1-F7}$	30.648	2.252	<0.001	36.499	1.473	<0.001
$\mu _\text {FP2-F4}$	39.058	2.107	<0.001	58.268	1.608	<0.001
$\mu _\text {FP2-F8}$	48.853	3.615	<0.001	54.765	1.947	<0.001

**Table 10:** Fitted $\hat{\varPi }$ matrices for the two periods

	$\hat{\varPi }$ prior to seizure				$\hat{\varPi }$ during seizure			
	FP1-F3	FP1-F7	FP2-F4	FP2-F8	FP1-F3	FP1-F7	FP2-F4	FP2-F8
FP1-F3	4.388	1.572	$-$11.120	5.743	$-$5.305	$-$11.021	9.447	0.971
FP1-F7	1.519	0.892	$-$2.985	0.725	$-$4.335	$-$7.285	6.275	1.116
FP2-F4	0.540	$-$0.050	$-$1.971	1.589	$-$10.265	$-$17.047	14.686	2.681
FP2-F8	$-$0.733	$-$1.658	$-$1.613	4.108	$-$14.907	$-$14.729	12.909	5.776

On the left side is the estimated matrix prior to the seizure, on the right side is the estimated matrix during the seizure

Here we can determine an all-to-all coupling during both periods and the estimated cointegration matrices show a clear difference for the two intervals. Prior to the seizure the right side signals FP2-F4 and FP2-F8 are much less influenced by the feedback in the system, whereas during the seizure both experience a much larger feedback from the left side signals FP1-F3 and FP1-F7 respectively. Surprisingly, the FP2-F8 signal does not seem to impose a large influence in the system in either interval. It is also interesting to note the changing signs in the two matrices. The two left side signals exhibit a positive feedback on themselves prior to the seizure, whereas during the seizure they impose a negative feedback both on themselves and the right side signals. This could possibly be part of an explanation of the slight kink seen in the phases around 3015–3020 s halfway through the seizure.

Concluding on this analysis we find, not surprisingly, a fully coupled 4 dimensional system with a clear change in the trends prior to and during the seizure. We find that during the seizure the interaction in the system is much stronger, suggesting the more distinctive phases shown in this interval. Including this temporal effect into a single cointegration model covering the full period by utilizing regime switching cointegration models, would be an interesting pursuit for future work.

## Discussion

In this paper we have investigated the use of cointegration analysis to determine coupling structures in linearly phase coupled systems. Using these techniques we can with a good precision identify the coupling structure as a subspace for this type of model. A standard measure to identify synchronization in the literature is the mean phase coherence measure. Contrary to this standard measure, we can detect uni-directional coupling, and we can construct and test hypotheses on the model in form of linear restrictions in the estimated subspace. Furthermore, comparing the mean phase coherence measure with the cointegration analysis in Sect. 4.6, we found that cointegration detects interaction in a system more robustly and for weaker coupling strength than does the mean phase coherence measure. Combined with the fact that cointegration does not just provide a level of synchronization, but rather the structure of the synchronization mechanism, this technique can be used to infer system structures in a much more detailed manner. Of course this higher level of information comes at a cost, since the mean phase coherence measure is easily implemented for any system, whereas the cointegration analysis is more involved and time consuming.

Due to the linear nature of the cointegration theory used, we are not able to cover more complex models, such as the Kuramoto model. Thus, an important extension for future work would be to allow for nonlinear coupling functions. However, the linear structure appears naturally when considering a linearization around some phase-locked state, such as for systems showing synchrony or asynchrony. Another interesting pursuit is to extend the model framework to include nonlinear deterministic trends, such that also models like the FitzHugh–Nagumo or the van der Pol oscillator would be covered. The model considered in this paper was constructed from the starting point of the phase process in the spirit of the Kuramoto model, and noise was added on this level. Another approach would be to start from a biological model or a reduction thereof and introduce the noise on the DGP. This would also lead to non-linearities both in drift and diffusion of the phase process. Finally, high dimensional systems are a major challenge in the area of coupled oscillators, hence it would only be natural to investigate cointegration properties of high dimensional systems. A system of more than two synchronizing oscillators that are nonlinearly phase coupled, facilitate chaotic behavior since phases can then bilaterally attract and repel each other. When the number of oscillators increase, one quickly ends up with intuitive shortcomings. The number of parameters rapidly increase with the dimension of the system, possibly leading to a desirable reduction to a sparse interaction structure. This is a key issue with the cointegration framework, which take into account all individual oscillators, as opposed to a mean-field approach that does not run into the same curse of dimensionality. The quality of the estimators will rapidly decrease with increasing dimension of the parameter space or numerical problems may arise. This problem might be alleviated by imposing a sparse interaction structure through a LASSO $L_1$ penalization.

Cointegration to identify coupling of oscillators has been attempted before in a neuroscience context by Dahlhaus and Neddermeyer (2012). There, the Kuramoto model is approximated for strongly phase coupled oscillators by setting $\sin (\phi _j - \phi _i) \approx \phi _j-\phi _i$, since the phase differences are assumed to be small. We have used the idea from Dahlhaus and Neddermeyer (2012) of analyzing the unwrapped multivariate phase process. Contrary to Dahlhaus and Neddermeyer (2012), however, we have not linearized the sine function to replicate Kuramoto, since this will cause a discrepancy when the phase difference of two oscillators is closer to $\pi$ than 0 (or $2\pi$). To mitigate this problem, we have instead taken the approach of designing a DGP with the properties we are interested in, and which allows for any phase differences. Furthermore, this DGP enables us to specify a cointegration model that comply with data from this DGP. Although it may not fully comply with a biological model, it can point to where necessary flexibility is needed in order to develop more realistic cointegration models for biological processes. A first attempt to analyze EEG signals with cointegration analysis with linear coupling structures has been presented. The results are promising, and reveal a finer dependence structure characterizing states of seizure and non-seizure in epileptic patients, which in this example was not possible from the simple Mean Phase Coherence measure. To fully explore the potential of the cointegration analysis for EEG signals, it would be useful to extend the model and analysis tools to allow for non-linearities and simultaneous treatment of many traces, as well as time varying coupling strengths.

Summing up, by applying cointegration as a technique to the field of coupled oscillators in biology, we open up for a whole new area of applications for this statistical theory. On the other hand, using cointegration methods, biologists can gain new insights into network structures, being able to fit models and carry out statistical hypothesis testing. If the cointegration framework presented in this paper can be extended to include the standard models currently used in the field, cointegration would prove a powerful analysis tool for researchers.

## Appendix 1: Derivation of an oscillating process with cointegrated phases

Using a transformation from polar to Cartesian coordinates, we can use Itô calculus to derive a data generating process $z_t=(x_{1t},y_{1t},\ldots ,x_{pt},y_{pt})'$, which will yield a phase process, $\phi _t$, and amplitude process, $\gamma _t$, that comply with (2) and (3).

Assume a system of *p* oscillators, such that $\phi _t\in {\mathbb {R}}^p$, $\gamma _t\in {\mathbb {R}}^p$ and $z_t\in {\mathbb {R}}^{2p}$. Let $\zeta _t = (\gamma _{1t},\phi _{1t},\ldots ,\gamma _{pt},\phi _{pt})'$ denote the 2*p*-dimensional process in the polar coordinates. For notational purposes, we omit the time index *t* for $z_t,\zeta _t,x_{kt},y_{kt},\phi _{kt}$ and $\gamma _{kt}$, where $k=1,\ldots ,p$, then we have the following relations

$$\begin{aligned} z = (x_1,y_1,\ldots , x_p,y_p)' = h(\zeta ) = \begin{pmatrix} \gamma _1\cos (\phi _1) \\ \gamma _1\sin (\phi _1) \\ \vdots \\ \gamma _p\cos (\phi _p) \\ \gamma _p\sin (\phi _p) \end{pmatrix}\in {\mathbb {R}}^{2p}. \end{aligned}$$

For $k=1,\ldots ,p$, we find that the *k*’th coordinate pair $(x_k,y_k)'$ are given as the $2k-1$ and 2*k* entries in $z=h(\zeta )$, i.e., $(x_k,y_k)' = (z_{2k-1},z_{2k})'=(h_{k-1}(\zeta ),h_k(\zeta ))'=(\gamma _k\cos (\phi _k),\gamma _k\sin (\phi _k))'$. Using the multivariate version of Itô’s Lemma, we find that for $k=1,\ldots ,p$

$$\begin{aligned} dz_{2k-1}&= \sum _i \frac{\partial \gamma _k\cos (\phi _k)}{\partial \zeta _i} d\zeta _i+\frac{1}{2}\sum _{i,j}\frac{\partial ^2\gamma _k\cos (\phi _k)}{\partial \zeta _i\partial \zeta _j}d\zeta _id\zeta _j \\&= \frac{\partial \gamma _k\cos (\phi _k)}{\partial \gamma _k}d\gamma _k +\frac{\partial \gamma _k\cos (\phi _k)}{\partial \phi _k}d\phi _k \\&\quad +\frac{1}{2}\Biggl (\frac{\partial ^2\gamma _k\cos (\phi _k)}{\partial \gamma _k^2} (d\gamma _k)^2+\frac{\partial ^2\gamma _k\cos (\phi _k)}{\partial \phi _k^2} (d\phi _k)^2+2\frac{\partial ^2\gamma _k\cos (\phi _k)}{\partial \gamma _k\partial \phi _k} (d\gamma _kd\phi _k)\Biggr ) \\&= \cos (\phi _k)d\gamma _k-\gamma _k\sin (\phi _k)d\phi _k-\frac{1}{2} \gamma _k\cos (\phi _k)(d\phi _k)^2-\sin (\phi _k)(d\gamma _kd\phi _k) \\&= x_k\gamma _k^{-1}d\gamma _k-y_kd\phi _k-\frac{1}{2}x_k(d\phi _k)^2-y_k \gamma _k^{-1}d\gamma _kd\phi _k\\ dz_{2k}&= \sum _i \frac{\partial \gamma _k\sin (\phi _k)}{\partial \zeta _i} d\zeta _i+\frac{1}{2}\sum _{i,j}\frac{\partial ^2\gamma _k\sin (\phi _k)}{\partial \zeta _i\partial \zeta _j}d\zeta _id\zeta _j \\&= \cdots \\&=\sin (\phi _k)d\gamma _k+\gamma _k\cos (\phi _k)d\phi _k-\frac{1}{2}\gamma _k \sin (\phi _k)(d\phi _k)^2+\cos (\phi _k)(d\gamma _kd\phi _k) \\&= y_k\gamma _k^{-1}d\gamma _k+x_kd\phi _k-\frac{1}{2}y_k(d\phi _k)^2+x_k \gamma _k^{-1}d\gamma _kd\phi _k. \end{aligned}$$

Note that $\gamma _k,\phi _k$ are both uni-variate processes.

35 $$\begin{aligned} d\begin{pmatrix} x_k \\ y_k \end{pmatrix}&= \begin{pmatrix} x_k \\ y_k \end{pmatrix} \gamma _k^{-1}d\gamma _k+ \begin{pmatrix} -y_k \\ \,x_k \end{pmatrix} d\phi _k -\frac{1}{2} \begin{pmatrix} x_k \\ y_k \end{pmatrix}(d\phi _k)^2+ \begin{pmatrix} x_k \\ y_k \end{pmatrix} \gamma _k^{-1}d\gamma _kd\phi _k \nonumber \\&= \begin{pmatrix} (1+d\phi _k)\gamma _k^{-1}d\gamma _k-\frac{1}{2}(d\phi _k)^2 &{} \quad -d\phi _k \\ d\phi _k &{} \quad (1+d\phi _k)\gamma _k^{-1}d\gamma _k-\frac{1}{2}(d\phi _k)^2 \end{pmatrix} \begin{pmatrix} x_k \\ y_k \end{pmatrix} \end{aligned}$$

Insert the relations

$$\begin{aligned} d\gamma _k&= g_k(\phi ,\gamma )dt+\sigma _k^\gamma dW_k^\gamma \\ d\phi _k&= f_k(\phi ,\gamma )dt+\sigma _k^\phi dW_k^\phi \\ (d\phi _k)^2&= \left( \sigma _k^\phi \right) ^2dt \\ d\gamma _kd\phi _k&= \sigma _k^\gamma \sigma _k^\phi dt\\ \gamma _k&= \sqrt{x_k^2+y_k^2} \end{aligned}$$

into (35) and obtain

36 $$\begin{aligned} d\begin{pmatrix} x_k \\ y_k \end{pmatrix}&= \underbrace{\begin{pmatrix} -\frac{1}{2}\left( \sigma _k^\phi \right) ^2 &{} \quad &{} -f_k(\phi ,\gamma ) \\ f_k(\phi ,\gamma ) &{} \quad &{} -\frac{1}{2}\left( \sigma _k^\phi \right) ^2 \end{pmatrix} \begin{pmatrix} x_k \\ y_k \end{pmatrix}dt+ \begin{pmatrix} 0 &{} -\sigma _k^\phi \\ \sigma _k^\phi &{} 0 \end{pmatrix} \begin{pmatrix} x_k \\ y_k \end{pmatrix}dW_k^\phi }_{\text {''Phase related''}} \nonumber \\&\quad + \underbrace{ \frac{g_k(\phi ,\gamma )+\sigma _k^\gamma \sigma _k^\phi }{\sqrt{x_k^2+y_k^2}} \begin{pmatrix} x_k \\ y_k \end{pmatrix}dt+ \frac{\sigma _k^\gamma }{\sqrt{x_k^2+y_k^2}} \begin{pmatrix} x_k \\ y_k \end{pmatrix}dW_k^\gamma }_{\text {''Amplitude related''}} \end{aligned}$$

The quotation marks in the description in (36) imply that one can intuitively interpret the system in this way, but the system is not mathematically split into these parts, as clearly *f* and *g* both depends on $\phi$ and $\gamma$, and $\sigma _k^\phi$ enters in the ”amplitude” part.

Generalizing (36) we find

37 $$\begin{aligned} dz_t = (R_t+Q_t)z_t dt+a(z_t,\varSigma _\phi )dW^\phi +b(z_t,\varSigma _\gamma )dW^\gamma , \end{aligned}$$

where $R_t$ is a block diagonal matrix of $2\times 2$ rotation matrices and $Q_t$ is a diagonal matrix of amplitude dependent adjustments. The noise is composed of a sum of two state dependent multivariate processes, where the functions *a*, *b* define the noise as given in (36). The time index *t* has been added in (37) to emphasize the time dependency of the matrices $R_t$ and $Q_t$.

## Appendix 2: Rank test for $\varPi$ and estimation of cointegrated models

Here we outline the procedure for determining the cointegration rank and estimating parameters in model (2). For a thorough presentation of this method, see Johansen (1996).

We refer to model (16) with ${{\mathrm{rank}}}(P)=r$ and $\mu _t=\mu$ as $H_r$. Using this categorization, we have a nested sequence of models

$$\begin{aligned} H_0 \subset \cdots \subset H_r \subset \cdots \subset H_r, \end{aligned}$$

which enables us to specify likelihood-ratio tests for the hypothesis $H_r$ given $H_{r+1}$ or $H_r$ given $H_p$, where $H_p$ is the unrestricted model. The first critical step for analyzing (2), is to determine the cointegration space dimension *r*. Given *r*, we can estimate the parameters in the model using *reduced rank regression* and ordinary least squares (OLS). The first step is to remove the deterministic trend by regression, then reduced rank regression is used to estimate *b* by solving an eigenvalue problem, and finally the remaining parameters are estimated by OLS, using the estimated $\hat{b}$.

To describe the regression procedure, some convenient notation needs to be established. Define for $n=1,\ldots ,N$, the following functions of the data, $\Upsilon _{0t_n} = \varDelta \phi _{t_n}, \Upsilon _{1t_n}=\phi _{t_{n-1}}$ and let $\varepsilon _{t_n}\sim \mathcal {N}_p(0,\varOmega )$.

The log-likelihood function is then (up to a constant)

38 $$\begin{aligned}&\log L(a, b, \mu , \varOmega ) = -\frac{1}{2}N\log |\varOmega |\nonumber \\&\quad -\frac{1}{2}\sum _{n=1}^N (\Upsilon _{0t_n}-ab' \Upsilon _{1t_n}-\mu )'\varOmega ^{-1}(\Upsilon _{0t_n}-ab' \Upsilon _{1t_n}-\mu ). \end{aligned}$$

Define

39 $$\begin{aligned} {\mathbb {R}}^{p\times p}\ni M_{ij}&= N^{-1}\sum _{n=1}^N \Upsilon _{it_n}\Upsilon '_{jt_n},\quad \text {for }i,j=0,1 \nonumber \\ {\mathbb {R}}^p\ni M_{i2}&= N^{-1}\sum _{n=1}^N \Upsilon _{it_n}, \quad \text {for }i=0,1 \nonumber \\ {\mathbb {R}}\ni M_{22}&= 1. \end{aligned}$$

Note that $M_{ij}=M'_{ji}$. Then the estimate of $\mu$ given *a* and *b* is

40 $$\begin{aligned} \hat{\mu }(a,b)&= M_{02}-ab{12} \nonumber \\&= N^{-1}\sum _{n=1}^N \bigl (\varDelta \phi _{t_n}-ab'\phi _{t_{n-1}}\bigr ) \end{aligned}$$

Define the residuals

41 $$\begin{aligned} \begin{aligned} R_{0t_n}&= \Upsilon _{0t_n}-M_{02} = \varDelta \phi _{t_n} - N^{-1}\sum _{n=1}^N \varDelta \phi _{t_n} \\ R_{1t_n}&= \Upsilon _{1t_n}-M_{12} = \phi _{t_{n-1}} - N^{-1}\sum _{n=1}^N \phi _{t_{n-1}} \end{aligned} \end{aligned}$$

With these preliminary steps, we obtain the profiled likelihood function

42 $$\begin{aligned} \log L(a, b, \varOmega ) = -\frac{1}{2}N\log |\varOmega |-\frac{1}{2}\sum _{n=1}^N (R_{0t_n}-ab' R_{1t_n})'\varOmega ^{-1}(R_{0t_n}-ab' R_{1t_n}), \end{aligned}$$

equivalent to the regression equation

43 $$\begin{aligned} R_{0t_n}=ab'R_{1t_n}+\hat{\varepsilon }_{t_n}. \end{aligned}$$

Equation (43) is estimated as a reduced rank regression, by solving for eigenvalues. Define

44 $$\begin{aligned} S_{ij} = N^{-1}\sum _{n=1}^N R_{it_n}R_{jt_n}' = M_{ij}-M_{i2}M_{2j},\quad \text {for }i,j=0,1. \end{aligned}$$

Then for a fixed *b*, we obtain estimates for *a* and $\varOmega$ by OLS with $b'R_{1t_n}$ as the independent variable,

45 $$\begin{aligned} \hat{a}(b)&= S_{01}b({b'S_{11}b})^{-1}, \end{aligned}$$

46 $$\begin{aligned} \hat{\varOmega }(b)&= S_{00}-S_{01}b({b'S_{11}b})^{-1}b'S_{10}, \end{aligned}$$

and the likelihood is then maximized as

$$\begin{aligned} L_\text {max}^{-2/N} (b) = |S_{00}-S_{01}b(b'S_{11}b)^{-1}b'S_{10}|. \end{aligned}$$

Using the Schur complement for the matrix

47 $$\begin{aligned} \begin{vmatrix} S_{00}&\quad S_{01}b \\ b'S_{10}&\quad b'S_{11}b \end{vmatrix}, \end{aligned}$$

we find

48 $$\begin{aligned} |S_{00}-S_{01}b(b'S_{11}b)^{-1}b'S_{10}| = |S_{00}|||b'(S_{11}-S_{10}S_{00}^{-1}S_{01})b|/|b'S_{11}b|. \end{aligned}$$

Equation (48) is maximized for all $p\times r$ matrices by solving for the *p* eigenvalues $\lambda _i$ in

49 $$\begin{aligned} |\lambda S_{11}-S_{10}S_{00}^{-1}S_{01}| = 0, \end{aligned}$$

such that

$$\begin{aligned} \lambda _i S_{11} v_i = S_{10}S_{00}^{-1}S_{01}v_i, \end{aligned}$$

and the $p\times 1$ eigenvectors $v_i, i=1,\ldots ,p$ are normalized,

50 $$\begin{aligned} v_j'S_{11}v_i = {\left\{ \begin{array}{ll} 1,\quad \text {for } i=j \\ 0,\quad \text {for } i\ne j. \end{array}\right. } \end{aligned}$$

Then for a given *r*, $\hat{b}$ ($p\times r$) is given by the *r* eigenvectors, $v_1,\ldots ,v_r$, corresponding to the *r* largest eigenvalues $\hat{\lambda }_1>\cdots >\hat{\lambda }_r$, and the maximum value of the likelihood function with this $\hat{b}$ is

51 $$\begin{aligned} L_\text {max}^{-2/N} =|S_{00}|\prod _{i=1}^r(1-\hat{\lambda }_i). \end{aligned}$$

Since (51) holds for $r=0,\ldots ,p$, where for $r=0$ we set $\text {sp}(\hat{b})=\{0\}$ and for $r=p$ we set $\text {sp}(\hat{b})={\mathbb {R}}^p$, we have solved for all possible ranks *r* once and for all, and we can form the likelihood ratio test

52 $$\begin{aligned} -2\log Q\bigl (H_r|H_p \bigr ) = -N\sum _{i=r+1}^p \log (1-\hat{\lambda }_i), \end{aligned}$$

for the model $H_r$ versus the model $H_p$. Equation (52) is known as the *trace statistic*, whereas

53 $$\begin{aligned} -2\log Q\bigl (H_r|H_{r+1} \bigr ) = -N\log (1-\hat{\lambda }_{r+1}), \end{aligned}$$

is known as the *maximum eigenvalue statistic*. The asymptotic distributions of (52) and (53) are both non-standard mixed Gaussian. Critical values for these can be found via simulation of a $p-r$ dimensional Brownian motion and using either the *trace* or *maximum eigenvalue* of a specially constructed $(p-r)\times (p-r)$ matrix, where the construction of the matrix depends on the structure of the deterministic terms in the model. Another possibility is to use *bootstrapping* as presented by Cavaliere et al. (2012).

Using the trace test (52), the rank *r* is then determined by proceeding as follows

1. Initialize with $$r=0$$.
2. For $r=0,\ldots , p-1$, if $H_r$ versus $H_p$ is rejected, set $r \rightarrow r+1$ and calculate (52).
3. Repeat step 2 until $H_r$ versus $H_p$ cannot be rejected, and set ${{\mathrm{rank}}}(\varPi )=r$.
4. If $$r+1=p$$, set $$r=p$$.

When the rank *r* is determined, then $\hat{b}$ is used for estimating the remaining parameters which follows from Eqs. (40), (45) and (46).

Observe some conveniences of Johansens procedure. First, all the *p* eigenvalues are determined at the same time. Thus, the eigenvalue problem only needs to be solved once through the whole procedure. Secondly, with tabulated or simulated values for the likelihood ratio tests, determining *r* follows a simple procedure. Finally with *b* fixed, the remaining parameters are estimated using OLS.

## Footnotes

1 The Hilbert transform of a signal $x_t$ is defined as $H(x_t) = \pi ^{-1}\text {p.v.}\int _{-\infty }^\infty \frac{x_\tau }{t-\tau }d\tau = -\pi ^{-1}\lim _{\varepsilon \rightarrow 0}\int _\varepsilon ^\infty \frac{x_{t+\tau }-x_{t-\tau }}{\tau }d\tau$, where $\text {p.v.}\int _{-\infty }^\infty$ denotes the principal value integral.
